# Aqueous Photolysis of Water-Soluble Brown Carbon from Simulated Prescribed and Wildfire Biomass Burning

**DOI:** 10.1021/acsestair.4c00016

**Published:** 2024-07-10

**Authors:** Mingrui Sun, Chase K. Glenn, Omar El Hajj, Kruthika V. Kumar, Anita Anosike, Robert Penland, Mac A. Callaham, E. Louise Loudermilk, Joseph J. O’Brien, Rawad Saleh, Geoffrey D. Smith

**Affiliations:** † Department of Chemistry, University of Georgia, Athens, Georgia 30602, United States; ‡ School of Environmental, Civil, Agricultural and Mechanical Engineering, University of Georgia, Athens, Georgia 30602, United States; § U.S. Department of Agriculture Forest Service, Southern Research Station, Athens Prescribed Fire Science Laboratory, Athens, Georgia 30602, United States

**Keywords:** brown carbon, water-soluble brown carbon, wildfire, prescribed burning, photobleaching, photo-enhancement, light absorption, duff

## Abstract

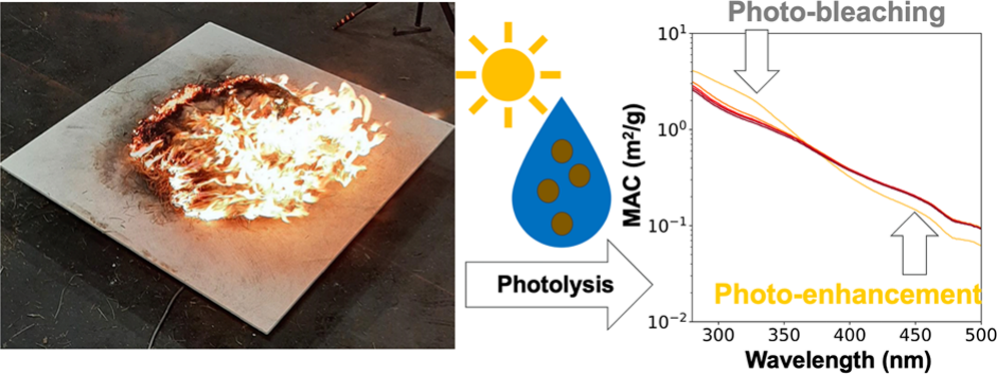

This work, as part of the Georgia Wildland fire Simulation Experiment (G-WISE) campaign, explores the aqueous photolysis of water-soluble brown carbon (W-BrC) in biomass burning aerosols from the combustion of fuel beds collected from three distinct ecoregions in Georgia: Piedmont, Coastal Plain, and Blue Ridge. Burns were conducted under conditions representative of wildfires, which are common unplanned occurrences in Southeastern forests (low fuel moisture content), and prescribed fires, which are commonly used in forest management (higher fuel moisture content). Upon exposure to radiation from UV lamps equivalent to approximately 5 h in the atmosphere, the absorption spectra of all six samples exhibited up to 40% photobleaching in the UV range (280–400 nm) and as much as 30% photo-enhancement in the visible range (400–500 nm). Together, these two effects reduced the absorption Ångström exponent (AAE), a measure of the wavelength dependence of the spectrum, from 6.0–7.9 before photolysis to 5.0–5.7 after. Electrospray ionization ultrahigh-resolution mass spectrometry analysis shows the potential formation of oligomeric chromophores due to aqueous photolysis. This work provides insight into the impacts that aqueous photolysis has on W-BrC in biomass burning aerosols and its dependence on fuel bed composition and moisture content.

## Abstract 2

This work studies the effect of UV photolysis on the absorption spectra and chemical composition of the water-soluble fraction of biomass burning aerosol generated from simulated wildfire and prescribed burning of fuel beds from three different ecoregions.

## 1 Introduction

Atmospheric aerosols possess the ability to alter incoming solar radiation through direct scattering or absorption of light. Among various aerosol species, brown carbon (BrC) is notable for its absorption of solar radiation, particularly within the UV region (280-400 nm), with the absorption capability diminishing at longer wavelengths, rendering its characteristic brownish color.^1^ As a potent light absorber, BrC has been estimated to be responsible for up to 24% of the aerosol warming effect and can influence tropospheric photochemistry by reducing the amount of solar UV radiation reaching the Earth’s surface.^2,3^ Upon emission into the atmosphere, the optical and chemical properties of BrC can continue to evolve through aging processes in aerosol particles and in aerosol liquid water (ALW), cloud, and fog droplets. The exact impacts of BrC on climate depend on the aging processes it undergoes and its composition.^4^ The present work explores specifically the role that photochemical aging plays in modifying the properties of BrC.

A principal source of atmospheric BrC is biomass burning, which occurs in forms such as open vegetation fires or anthropogenic burning of biofuels. It has been estimated that biomass burning contributes approximately 85% of global primary organic aerosol production and accounts for 60% of the warming effect associated with BrC.^5,6^ The optical properties of biomass burning BrC exhibit a high degree of variability, depending on the fuel type and combustion conditions. Currently, biomass burning BrC contributes to uncertainty in climate models, attributed to the limited understanding of its sources, aging processes, and optical properties.^7^ The present work explores two factors that have been insufficiently studied with regard to biomass burning aerosol optical properties: (1) prescribed-fire versus wildfire combustion conditions and (2) duff burning.

Prescribed burning is a forest management strategy aimed, in part, at mitigating the escalating economic and ecological risks posed by wildfires. From a combustion standpoint, the main distinction between wildfire and prescribed fire lies in the amount of moisture content in the fuels; prescribed fires are conducted under relatively high moisture content fuel conditions, especially following rainfall, while the majority of burned areas consumed by wildfires occur under drier fuel conditions such as during drought events. In the Southeastern U.S., most forest fires are prescribed fires as opposed to wildfires.^8^ Due to the prevalence of prescribed burning, it is important to assess its aerosol production and the properties of the emitted aerosol so as to reduce its impact and inform fire management policy.

In some areas of the Southeastern U.S., the duff layer can contribute significantly to biomass burning fires.^9^ Duff, originating from detritus or decomposed plant organic material, forms a layer of combustible organic material atop the mineral horizons of the soil, and such accumulations of organic material are commonly seen in the forest floor of various forest types across North America, Europe, and Asia.^10^ In locales with warm, moist climates and extensive forest cover, such as the southern Appalachians, deep duff can be formed, particularly in forest types dominated by plants that produce more recalcitrant leaf litter, which results in slower decomposition rates of organic material.^11,12^ Duff materials exhibit distinct burning emissions and flammability characteristics compared to other fuels.^8^ Significant burning of duff occurs only in dry conditions, such as in wildfires following prolonged droughts, due to its limited flammability in moist conditions.^11^ With the increasing frequency of extreme drought,^13,14^ the importance of duff combustion on the climate may only increase.^8^

Liquid water in the atmosphere acts as a crucial medium for various reactions that modify the chemical composition and optical properties of atmospheric aerosols.^4^ Specifically, the aqueous photolysis process, including direct photolysis and secondary processes like hydroxyl radical (OH) photo-oxidation, can transform organic aerosol within ALW, cloud, or fog droplets. This transformation may result in either decreased absorption (photobleaching) or increased absorption due to the creation of new chromophores (photo-enhancement).^4^ The photo-enhancement effect has been observed from the UV exposure of various substances, including water-soluble BrC from wood smoke,^15,16^ ambient biomass burning BrC,^15^ BrC from the burning of urban construction material,^17^ and secondary BrC surrogates (nitrophenols).^18^ Size exclusion chromatography studies have demonstrated that the photo-enhancement effect originates from the formation of larger chromophore species during the photochemistry process.^15^ A recent study indicated that woody fuel (e.g., pine wood) smoke exhibits a more pronounced photo-enhancement effect compared to dung cake, which primarily demonstrates photobleaching under aqueous photolysis. This observation was attributed to the fact that woody fuel smoke contains more monoaromatic species^19,20^ that are likely to form larger chromophores via oligomerization.^4,19^ Despite considerable research, the impact of aqueous photolysis on fresh biomass burning BrC from real-world sources like wildfires remains unclear, largely because most studies have utilized furnace-generated aerosols, which may not accurately mimic the conditions and chemical composition of wildfires.^13,14,16^

In this study, we explore the effects of aqueous photolysis on water-soluble biomass burning BrC collected from the G-WISE (Georgia Wildland-fire Simulation Experiment) campaign simulating both prescribed burns and wildfires. Fuel bed materials, collected from three distinct forest regions of Georgia (Piedmont, Coastal Plain, and Blue Ridge), were combusted under conditions simulating both prescribed burns (higher moisture content) and wildfires (low moisture content) to investigate variations due to source and burning conditions. Notably, the Blue Ridge sample contained a significant fraction of the duff. During photolysis, the UV-vis absorption spectra of the samples were collected, and their chemical compositions were analyzed using electrospray ionization ultrahigh-resolution mass spectrometry (ESI-UHR-MS).

## 2 Material and Methods

### 2.1 Biomass Burning Aerosol Generation in the G-WISE Campaign

The G-WISE campaign was conducted at the U.S. Forest Service Prescribed Fire Science Laboratory (U.S. Forest Service Southern Research Station, GA, USA) from October 25, 2022 to November 19, 2022. The fuels used in this study were sourced from the Oconee National Forest (Piedmont), Chattahoochee National Forest (Blue Ridge), and Fort Stewart Military Reservation (Coastal Plain) in Georgia. At each location, fine and litter fuels were collected with rakes, while woody fuels were handpicked. These fuels were placed into separate bags and later categorized based on their fuel types. After collection, the fuels were conditioned to the desired moisture levels and monitored using a fuel moisture analyzer (Computrack 4000 XL, Brookfield Ametek, MA), through methods such as oven drying, water submersion, or humidifier room placement, depending on the fuel type. Upon achieving the desired moisture levels, portions of the fuels were weighed and stored in zip-lock bags until the day of burning.

In this work, two fuel moisture levels were examined: higher moisture, which is more representative of prescribed burns, and lower moisture, which is more representative of wildfire burns. The moisture content of the lower-moisture (wildfire) fuel beds was not adjusted after the fuels were dried and weighed and was 2–3% by weight. The higher-moisture (prescribed fire) fuel bed components were humidified by submerging in water and then dried to the desired moisture content (woody fuels) or placed in a walk-in humidifier until the target moisture content was reached (fine fuels). The moisture content of the duff component of prescribed Blue Ridge fuel beds was left as it was collected. The overall moisture content was 10–12% for Piedmont and Coastal Plain prescribed fuel beds and 50–60% for Blue Ridge prescribed fuel beds, since the duff component retained a significant amount of moisture.

**Figure 1:**
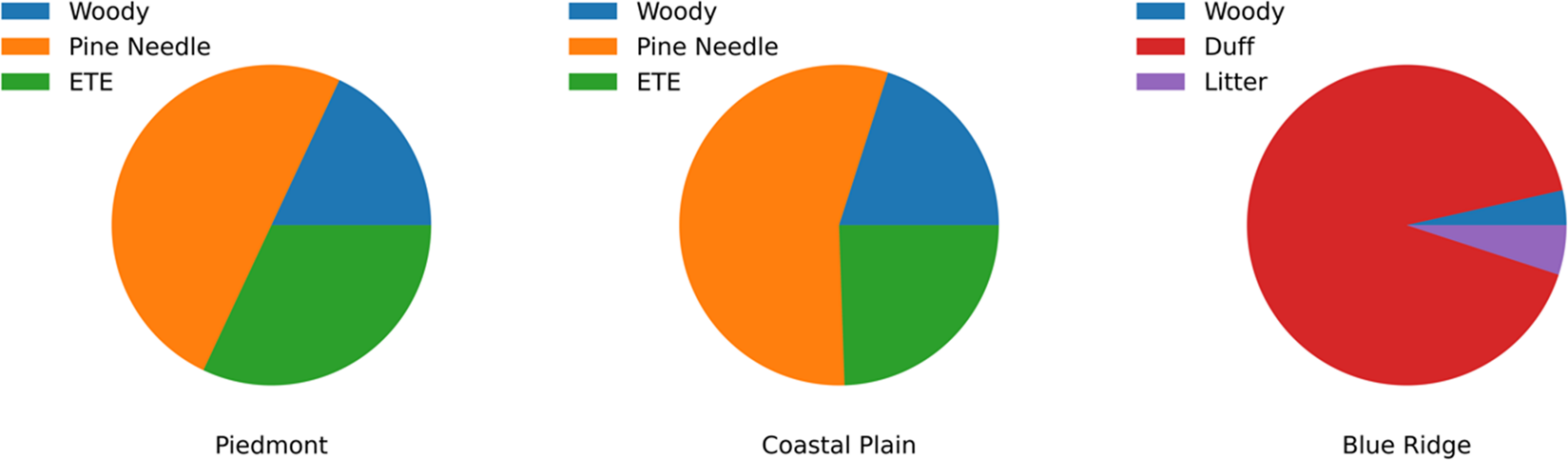
Composition breakdown (by mass) of fuel beds burned in this work. ETE stands for “everything else”, which includes grasses, forbs (herbaceous flowing plants that are not grasses, sedges, or rushes), and herbs.

On the burn day, a fuel bed was constructed based on the mass percentage of individual dry fuel types found in each ecoregion, and the fuel bed was assembled within a ring to mimic a natural fuel bed arrangement in the burn room (990 m^3^). The compositions of the fuel beds from the three different ecoregions (by mass) are illustrated in Figure 1. Ignition of the fuel bed was carried out under a simulated wind condition of approximately 1 meter/second generated by a bank of fans. Throughout the burning process, fire dynamics were monitored using an overhead a radiometric thermal imager (FLIR A655sc, Teledyne, OR) placed above the fuel bed ring. Once the smoldering phase of the burns concluded, as confirmed by the fuel bed temperature measured by the thermal imager dropping below 573 K, fresh smoke aerosols were collected on 47 mm PTFE filters (0.2 micron, Sterlitech Corporation) by directly sampling air from the burning room with no explicit particle size cut employed. The duration of collection for each filter ranged between 5 to 15 min depending on the specific aerosol concentration levels in the burn room. Filter samples were stored in sterilized Petri dishes (Analyslide, PALL) in a refrigerator until extraction.

For each fuel source and burn condition, water-soluble particulate matter was extracted using the following procedure: 1) Two Teflon filters collected on the same day were extracted in 10 mL of methanol (Sigma-Aldrich, ACS spectrophotometric grade) in a glass vial under sonication for 30 min. 2) The methanol extracts were dried under N_2_ flow to concentrate the organic aerosol material in the vials. 3) 5 mL of 18.2 MΩ Milli-Q water was added to extract the water-soluble portion using sonication for 30 min. 4) The resulting sample was filtered with a 13 mm PTFE disposable syringe filter (0.2 μm, Omicron Scientific) to remove suspended insoluble materials. The pH of each extracted solution was measured using a pH meter (PHH222, Omega) and found to lie in the range of 7.6–8.5. The organic carbon (OC) concentration of each water extract was determined with a OCEC analyzer (Model 5 L, Sunset Laboratory Inc.) following the NIOSH-870 protocol.^21^ These concentrations range from 30 to 70 μg/cm^3^ and are much larger than typical concentrations in atmospheric water, which can range from 0.001 to 1 μg/cm^3^, and may be more similar to the higher concentrations that can be found in the liquid layers of deliquescent particles.^22,23^ A summary of the measured OC concentrations is shown in Table S1. Photolysis experiments and subsequent UV-vis and ESI-UHR-MS analyses were performed within 3 days following extraction. While only one burn per fuel bed type and burning condition combination was examined, the burning conditions were consistent for the three repeated burns of each combination.

### 2.2 Aqueous Photolysis Experiments

Photolysis experiments were carried out in a 3.5 mL quartz cuvette (10 mm light path, CV10Q3500F, Thorlabs) located inside a photoreactor (LZC photoreactor, Luzchem Research). The photoreactor was equipped with 16 UV lamps (RPR-3000A, S. N. E. Ultraviolet Corp) with emission from 290 to 340 nm (60% UVB/40% UVA), as measured by a spectroradiometer (RPS900, International Light Technologies). We focus on this region of the spectrum because these wavelengths have been found to be effective at promoting photobleaching and photo-enhancement. In particular, Choudhary et al. (2023) demonstrated significant photobleaching with both UVA and UVB radiation, but photo-enhancement only with UVB radiation.^19^ The integrated flux on the cuvette setup was determined with an azoxybenzene actinometer following the protocol from Lignell et al. (2013).^24^ Samples were exposed to the lamp radiation for 2 h. By comparing the integrated (290-340 nm) flux measured for the lamps to the calculated integrated actinic flux over the same range of wavelengths for conditions representative of Athens, Georgia on 07/15/2023 using the “Quick TUV” online calculator,^25^ we estimate that the 2 h exposure is equivalent to 5 h of diurnally-averaged exposure in the atmosphere. Details of the emission spectrum, chemical actinometer measurement, parameters used in the “Quick TUV” calculator, and the solar condition scaling can be found in the Supporting Information, Figures S1–S6 and Table S2. Periodically during the 2 h photolysis process, the UV-vis spectrum of the W-BrC solution was measured at specific intervals: every minute (1–10 min), then every 5 min (15–30 min), then at 45, 60, 90, and 120 min. At these same times, 50 μL of the solution was removed for later ESI-UHR-MS analysis. It is important to point out, as have others,^4,16^ that it is difficult to differentiate direct photolysis from secondary photo-initiated reactions, such as OH photo-oxidation; in the present work, we do not make any attempt to distinguish these two mechanisms and, instead, examine the combined effects of both.

### 2.3 UV-Vis Spectrometer Measurements

Light absorption spectra during photolysis were measured on a double beam UV-Vis Spectrometer (Agilent, Cary 60) from 280 to 800 at 1 nm resolution. The effect of photo-enhancement or photobleaching was quantified by calculating integrated absorption in the UV (280–400 nm) and visible (400–500 nm) ranges. The raw UV-Vis spectrum was converted into mass absorption coefficients (MAC) in unit of m^2^/g from the base-10 absorbance (Abs_10_(λ)), OC concentration (g/m^3^), a 1 cm light path, *b*, using the following eq 1):
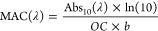
1The absorption Ångström exponent (AAE) of each sample was calculated as the negative slope of a linear fit of UV-vis absorption spectrum in a log-log plot over the 280–500 nm range. The upper limit of 500 nm was used for the fit because the absorption approached zero at longer wavelengths.

### 2.4 ESI(−)-UHR-MS Analysis

Mass spectral composition analysis was carried out on filter extracts before, in the middle of, and at the conclusion of each two h photolysis experiment for each fuel type and burn condition (wildfire or prescribed) combination. The middle point of photolysis was chosen to be near the peak of the photo-enhancement effect based on the UV-Vis absorption spectrum and varied by sample from 20 to 60 min of exposure. ESI-UHR-MS (electrospray ionization-ultrahigh-resolution-mass spectrometry) analysis was performed on a Bruker SolariX XR 12 T Fourier-Transform Ion Cyclotron Resonance (FT-ICR) mass spectrometer in negative mode over the 100–600 *m*/*z* range. The transient length was 0.5592 s, which yielded a resolution of 150000 at 400 *m*/*z*. External mass calibration was performed using sodium trifluoroacetate (NaTFA). Spectra for each sample were acquired at 48 scans averaged per spectra. Peak assignment of the resulting mass spectra was performed using the open-source R package MFassignR.^26^ For each mass spectrum, sample noise was removed using the KMDNoise function in the MFassignR package with a signal-to-noise cutoff of 3.^18^ Peak assignments were extracted following the MFAssignR isotope filtering and internal mass calibration steps. Assignments in this work were all done with elemental constraints of O ≤ 40, N ≤ 3, S ≤1, mass error tolerance of <1 ppm and limited to singly-charged species. Background subtraction was performed by removing peaks assigned in a water blank processed in the same way as the samples were. Mass error of two common biomass burning tracers, levoglucosan (C_6_H_10_O_5_) and vanillic acid (C_8_H_8_O_4_), was less than 0.3 ppm for all mass spectra obtained.

After formula assignment, O/C and H/C ratios and double bond equivalent (DBE) values^27^ were calculated for all formulas identified in each mass spectrum. DBE is equal to the sum of C=C and C=O bonds and rings in a molecule and is calculated from the number of C, H, and N atoms in a given formula:
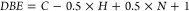
2where C, H, and N represent the number of the respective carbon, hydrogen, or nitrogen atoms in the formula.

## 3 Results and Discussion

### 3.1 Changes in W-BrC UV-Vis Absorption Spectra under Aqueous Photolysis

**Figure 2:**
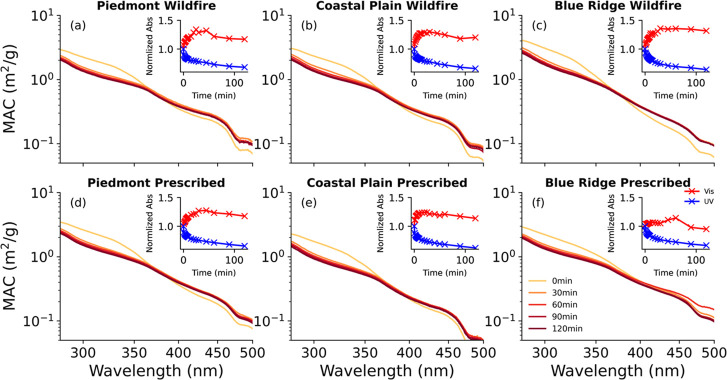
Absorption spectra progression (0–120 min) under direct aqueous photolysis with UV radiation for W-BrC samples from Piedmont (a, d), Coastal Plain (b, e), and Blue Ridge (c, f) fuel beds. Inset plots show the progression of integrated absorption (normalized to the 0 min value) in the UV (280–400 nm; blue symbols) and visible (400–500 nm; red symbols) regions.

We measured the W-BrC UV-Vis absorption spectra of particulate matter generated from the combustion of fuels from the three ecoregions and under both combustion conditions (wildfire and prescribed fire; Figure 2). The UV-vis spectra of the W-BrC are shown as a function of exposure (up to 2 h) to the UV light. The absorption spectra are generally featureless, consistent with literature reports of biomass burning aerosol.^28^ In general, all spectra follow a power law functional form, indicated by a straight line on the log–log plot, which is typical for BrC.^28^

During the photolysis process, photobleaching in the UV (280–400 nm) and photo-enhancement in the visible (400–500 nm) can be observed in all samples. The inset in each plot displays the normalized integrated absorption in the UV (280–400 nm; blue symbols) and the visible (400–500 nm; red symbols) regions of the spectrum as a function of exposure time to the UV light. Most of the UV photobleaching and the visible photo-enhancement effects occur within the first 20 min of exposure for each sample. The UV photobleaching tends to increase continuously with exposure time, with absorption decreasing by as much as 36%, while the visible photo-enhancement reaches a maximum (32–38%) and then decreases slowly with longer exposure time. The notable exception is the Blue Ridge prescribed sample, which demonstrates a much smaller peak photo-enhancement (14%). Similar levels of photobleaching over 2 h of exposure have been reported for W-BrC from BBAs originating from the burning of dung cakes in Choudhary et al. (2023).^19^ The fact that both photobleaching and photo-enhancement persist, even after 2 h, suggests that these effects may remain significant in ambient aerosols over longer time scales.

**Figure 3:**
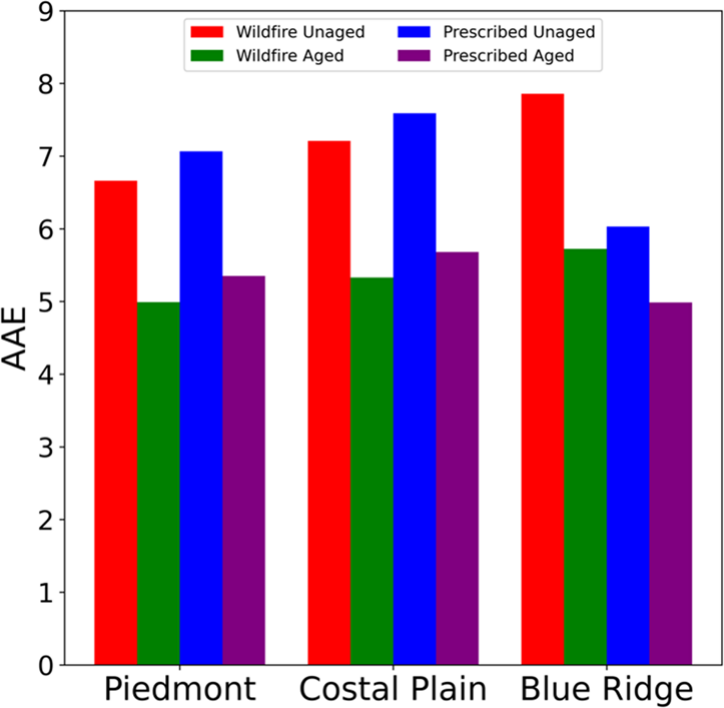
Absorption Ångström exponents (AAEs) measured for aqueous W-BrC in unaged samples (red and blue bars) and samples exposed for 2 h to UV light (green and purple bars).

The net effect of the UV photobleaching and visible photo-enhancement is a decrease in overall absorbance since the absorbance at UV wavelengths is much larger than at visible wavelengths. Consequently, the shape of each spectrum changes, as reflected by the values of the AAEs; unaged samples have AAE values ranging from 6.0 to 7.9, while aged samples have values of 5.0 to 5.7. The AAE values for both unaged and aged samples from the three ecoregions and under both wildfire and prescribed-fire conditions are shown in Figure 3. All AAE values lie within the weakly-absorbing BrC range defined by Saleh et al. (2020).^7^

The photolytic aging tends to result in spectra that have very similar spectral shapes despite initial differences according to fuel ecoregion and combustion condition. For the unaged Piedmont and Coastal Plain samples, the combustion conditions do not influence the value of the AAE much. On the other hand, the Blue Ridge samples show a noticeable difference in AAE of 7.85 (wildfire) vs 6.03 (prescribed). Since the Blue Ridge fuel bed is the only one that contained duff in this experiment, and since the duff does not combust under prescribed conditions, this observation indicates that the combustion of duff has an important influence on the composition of BrC and especially the absorption spectrum of the W-BrC.

Previous studies have also observed the photo-enhancement effect from the UV aqueous photolysis of furnace-generated biomass burning W-BrC with the increase in absorbance being quantified at single wavelengths (365 or 400 nm; summarized in Table S3), as opposed to the entire spectrum.^16,19,29^ To more directly compare our findings to those of these studies, we quantified the change in absorption that we observed at these two specific wavelengths (shown in Figure S7 and listed in Table S3). At 365 nm, we observe a photo-enhancement of 10–35%, which is similar to observations from the previous studies (39–68%).^16,19^ However, at 400 nm we observe only 5–20 % photo-enhancement compared to 150–235% observed in the previous studies.^16,19,29^ Three factors may contribute to the differences observed: (1) The biomass burning W-BrC generated in a furnace at a set temperature might inherently differ from that produced in more realistic open fire scenarios, as simulated in the present study. (2) The mixture of a variety of fuels used in each burn in the present study is broader compared to the exclusive use of firewood in the furnace studies, potentially influencing the resulting W-BrC composition. (3) The wavelength and intensity of lamps employed for photolysis in the present study differed from those used in the preceding research, which could lead to differences in the chemistry during the aqueous photolysis process. Collectively, these distinctions underscore the importance of examining aerosols generated in settings that closely mimic real-world forest fires to gain a more accurate understanding of their behavior and implications.

### 3.2 Chemical Composition Analysis of the Aqueous Photolysis Process

#### 3.2.1 Elemental Composition Changes with Photolysis

**Figure 4:**
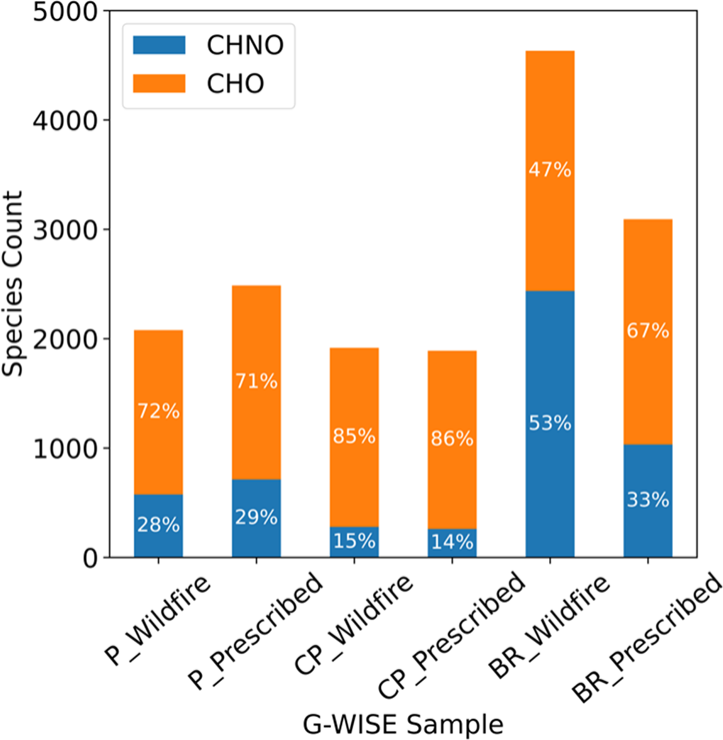
Formula distribution of each unaged sample ESI-UHR-MS mass spectrum. Formulae detected and assigned are almost entirely CHO and CHNO. P, CP, and BR stand for Piedmont, Coastal Plain, and Blue Ridge fuel beds, respectively.

To investigate chemical evolution through direct aqueous photolysis, an offline ESI-UHR-MS analysis was performed. The elemental group distribution of each unaged sample derived from these mass spectra are summarized in Figure 4. The formulae assigned are almost all of the form CHO or CHNO with a small fraction (< 0.1%) of the form CH or CHN. Significantly more molecular assignments were made from the Blue Ridge wildfire sample (4634 species) and Blue Ridge prescribed sample (3093 species) compared to the other four samples, each of which has approximately 2000 species. Additionally, the Blue Ridge wildfire sample stood out with the highest proportion of nitrogen-containing species, comprising 53% of the peaks, a stark contrast to the 14–29% observed with the other fuel beds. This observation suggests an increased level of chemical complexity, which is likely attributed to the combustion of duff material, setting it apart from other fuel types. Duff layers are a vital component of the soil nitrogen cycle, playing a key role in fixing and storing nitrogen content within forest soils.^30^ Consequently, when duff material burns, it likely contributes to the release of nitrogen-containing species. Conversely, duff does not combust under moist (prescribed) conditions, and for the Blue Ridge prescribed sample, we observe that there is a much lower fraction of nitrogen-containing species (33%). The underlying mechanisms leading to more nitrogen-containing W-BrC in association with duff burning remain unresolved, and this area of study requires further research to elucidate the specific processes and conditions contributing to these observed patterns.

**Figure 5:**
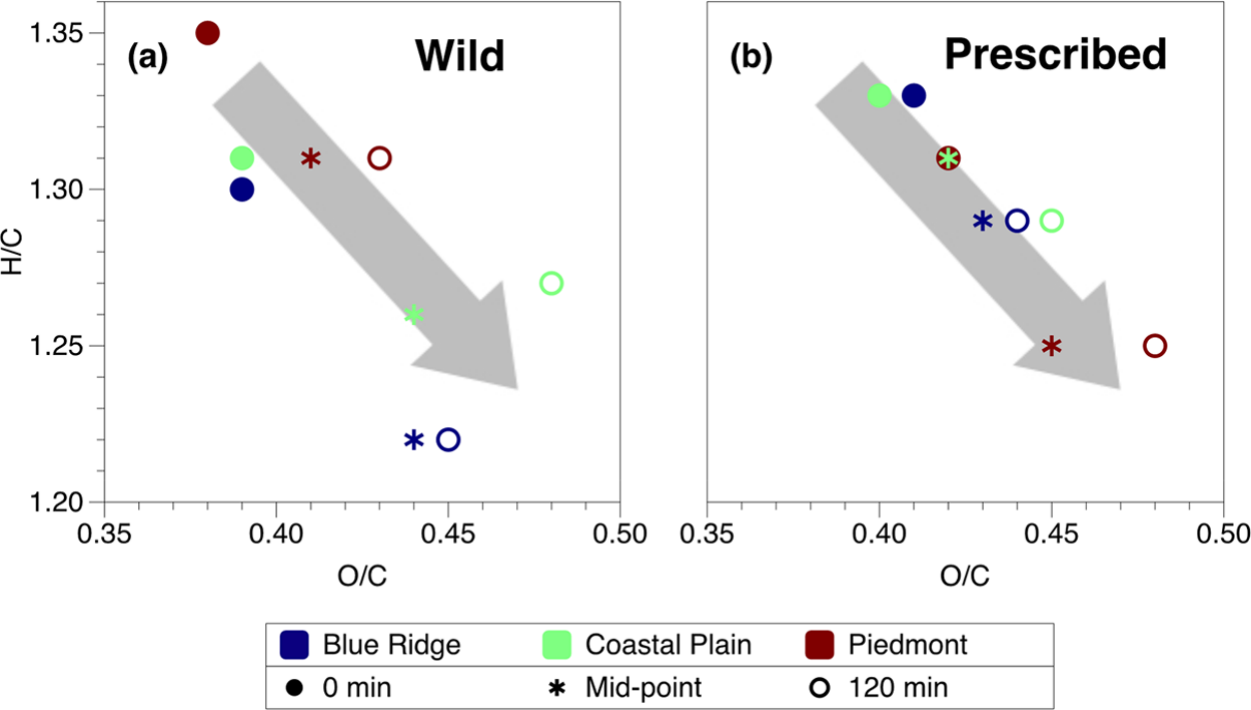
Van Krevelen plots of intensity-weighted average composition before exposure to UV light (solid circle; 0 min) and at the midpoint (star) and end (open circle; 120 min) of exposure. (a) and (b) show wildfire and prescribed-fire conditions, respectively. Overall, the composition of the samples evolves with exposure to the UV light, with the value of the O/C ratio increasing and the value of the H/C ratio decreasing.

The abundance-weighted average of the oxygen-to-carbon (O/C) and hydrogen-to-carbon (H/C) ratios of each sample is shown in a Van Krevelen plot in Figure 5. There are slight differences in these ratios for samples from the three different fuel types, but they exhibit the same general trend upon photolysis, with the O/C ratio increasing from approximately 0.38 to 0.48 and the H/C ratio decreasing from approximately 1.35 to 1.22. Though the changes in elemental composition are small, they are consistent with oxidation of the samples.^31^ Most of the change in composition is seen to occur by the mid-point, i.e., the time at which the photo-enhancement in the visible region of the spectrum has reached its maximum (see Figure 2). This correlation suggests that the inferred increase in oxidation may explain, at least in part, the increase in the absorption. We explore this hypothesis further in the next section, where we examine the changes in the ESI-UHR mass spectra upon photolysis in greater detail.

#### 3.2.2 Evidence for Potential Oligomerization with Photolysis

Previously, the photo-enhancement effect observed for biomass burning W-BrC has been studied using size exclusion HPLC (high-performance liquid chromatography) with UV-vis spectroscopy.^16^ In that study, photo-enhancement was attributed to the formation of long-lived, larger molecular species generated through oligomerization/polymerization or functionalization.^16^ Here, we investigate evidence for potential oligomerization occurring in the G-WISE W-BrC samples.

**Figure 6:**
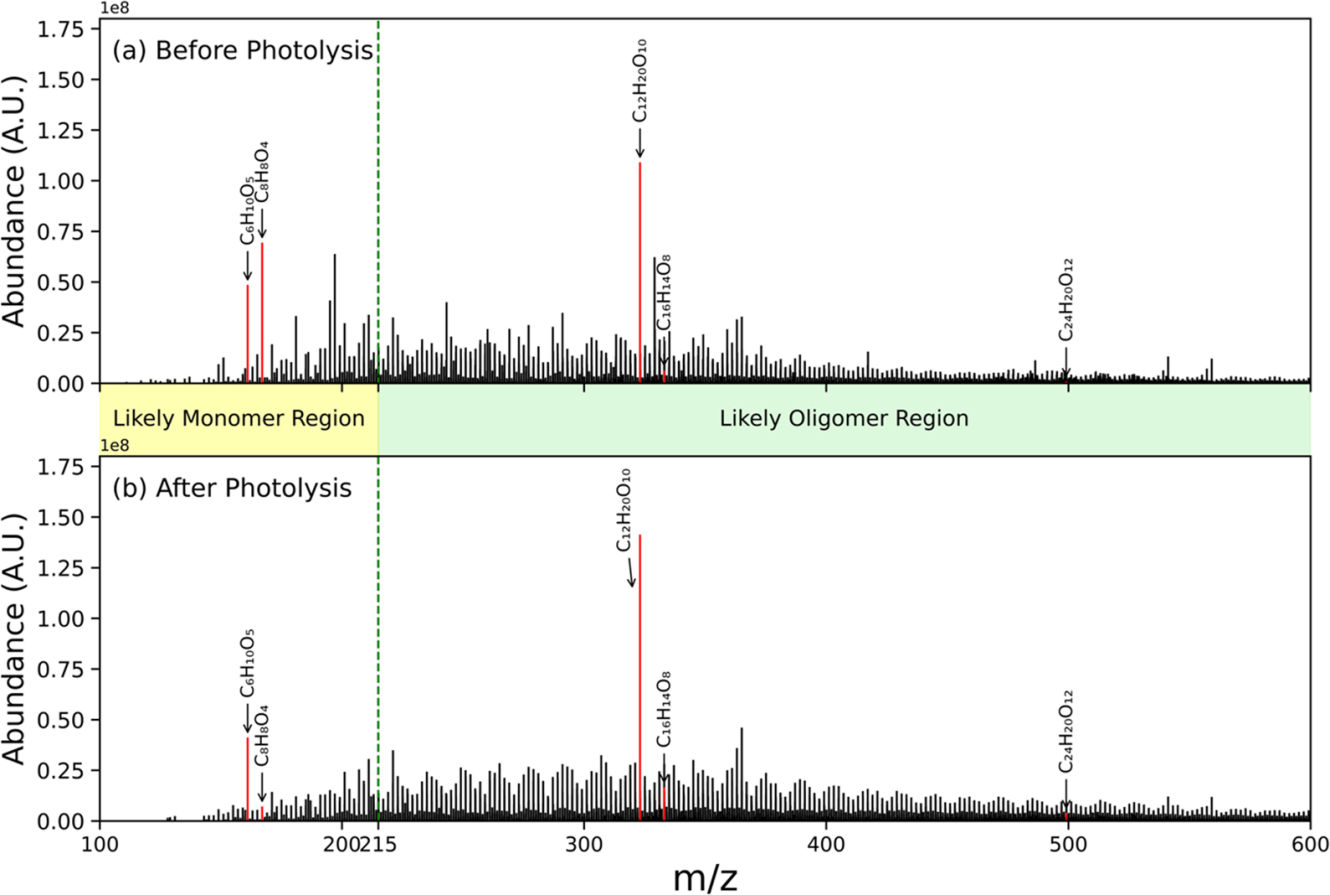
ESI-UHR-MS mass spectra of an aqueous extract of particulate matter generated from combustion of a Blue Ridge fuel bed under wildfire conditions (a) before and (b) after a 2 h aqueous UV photolysis. A more regular pattern of repeating units, suggesting oligomerization, can be seen in the mass spectrum after photolysis. Assigned peaks C_6_H_10_O_5_ (levoglucosan), C_8_H_8_O_4_ (vanillic acid), C_12_H_20_O_10_ (possible levoglucosan dimer), C_16_H_14_O_8_ (possible vanillic acid dimer), and C_24_H_20_O_12_ (possible vanillic acid trimer) are highlighted (in red) here as an example of specific oligomer production.

An example of how the mass spectra of the samples change upon exposure to UV light is shown in Figure 6 for a Blue Ridge wildfire sample. A pronounced formation of a regular pattern in the >215 *m*/*z* region following photolysis is seen with clusters of signals separated by 12, 14, 16, or 18 u, and notable peaks within each cluster separated by 2 u. This emergence of a regular pattern with repeating units is consistent with the formation of oligomers during photolysis.^32^ The emergence of similar patterns after photolysis is also observed for the other samples (Figures S9–S14).

**Figure 7:**
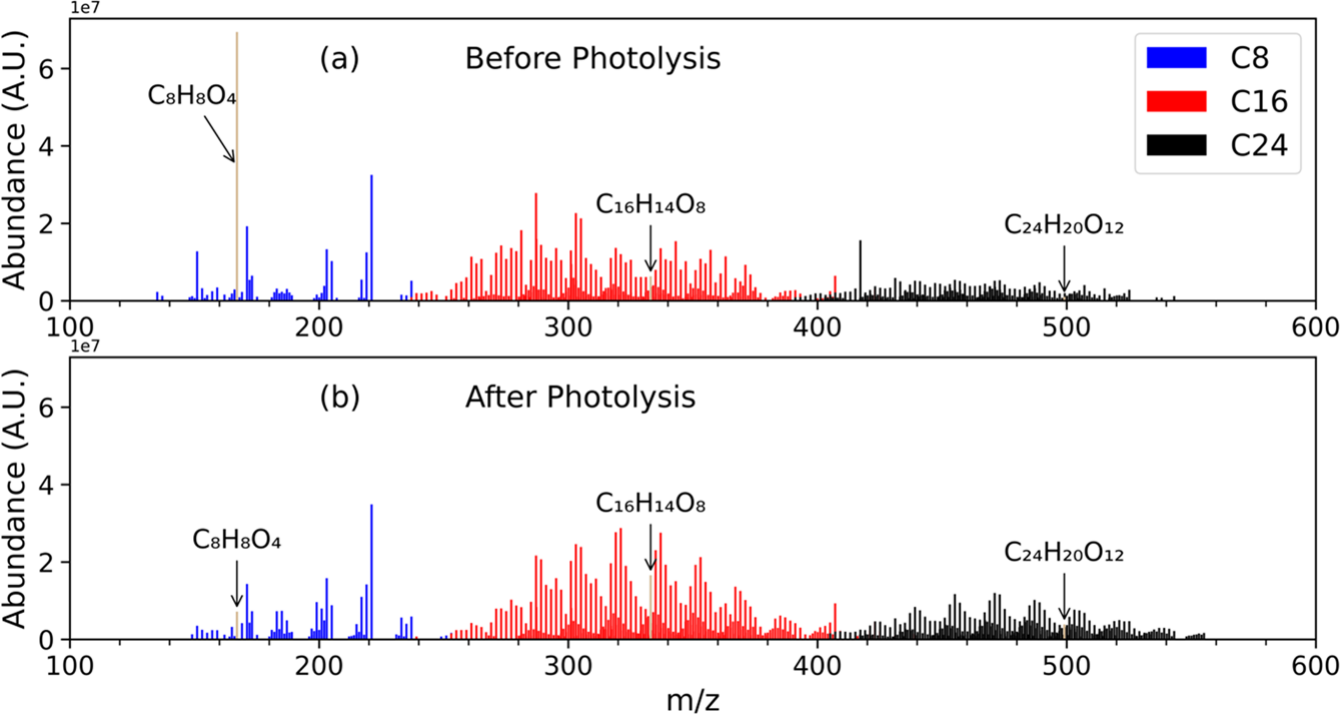
Reconstructed ESI-UHR-MS spectra of water-soluble particulate matter from the Blue Ridge fuel bed combusted under wildfire conditions focusing on just C_8_, C_16_, and C_24_ species. C_8_H_8_O_4_ (vanillic acid) and its dimer (C_16_H_14_O_8_) and trimer (C_24_H_20_O_12_) are highlighted in tan. After photolysis, an increase in the intensity of peaks in the likely dimer (C16) and trimer (C24) clusters is observed.

Furthermore, two monomers known to be components of biomass burning particles, vanillic acid (C_8_H_8_O_4_) and levoglucosan (C_6_H_10_O_5_),^33,34^ along with their respective oligomers, are highlighted in red in Figure 6. A significant suppression of the highlighted monomer signals and an enhancement of the oligomer signals postphotolysis are observed. While it is possible that the formation of dimers and trimers could occur as a result of ion clustering in the mass spectrometer, additional tests in which levoglucosan was added to the sample matrix indicate that this is not likely (see Figure S14). What is more, Tang et al.^35^ also studied the aqueous photo-oxidation of vanillic acid and observed a similar loss of the monomer and appearance of the dimer with accompanying photobleaching (λ < 320 nm) and photo-enhancement (λ > 320 nm). In their experiments, however, H_2_O_2_ was added as an OH precursor, and in the absence of it they saw negligible loss of the vanillic acid monomer indicating that it was the production of OH that was responsible for the dimerization they observed.^35^ The fact that we observe a significant depletion of the monomer upon exposure to UV light suggests that OH radicals might be generated and be responsible for the oligomerization. However, we are not able to distinguish such reactions from direct photolysis in our experiments. The depletion of the vanillic acid monomer and the emergence of potential dimer and trimer peaks as well as the regular pattern in the peaks is even more clearly illustrated in Figure 7 in which we plot only peaks corresponding to C_8_, C_16_, and C_24_ assignments.

Additionally, to illustrate quantitatively the increase in oligomer signal intensity, the oligomer-to-monomer ratio was calculated by dividing the integrated signal from the “oligomer” region (215–600 *m*/*z*) by that of the lower mass “monomer” region (100–215 *m*/*z*) of the full spectrum (i.e., Figure 6). Though we cannot conclusively assign all peaks in these regions as oligomers or monomers, this approach has been used previously,^19^ and the ratio allows us to measure changes occurring with photolysis that are consistent with what would be expected with oligomerization. A summary of the ratios from all of the tested samples is provided in Table 1. For all fuel beds and under both wildfire and prescribed fire conditions, this ratio increases significantly with photolysis with most of the increase occurring by the mid-point, corresponding to the peak of the photo-enhancement effect (see insets in Figure 2). The Blue Ridge samples exhibited a larger increase in the oligomer-to-monomer ratio (100% for wildfire and 52% for prescribed) than observed for the other samples (10–42%). It is not clear why the increase was so much larger, but it may be attributed to the inherently higher chemical complexity of Blue Ridge samples, for which more peaks were assigned (4634 for wildfire, 3093 for prescribed) than for the other samples (∼2000). Perhaps this increased complexity leads to more varied oligomerization, resulting in a higher oligomer-to-monomer ratio, but the underlying mechanisms responsible for this difference remain unresolved.

**Table 1:** Oligomer to Monomer Signal Ratio and BrC Chromophore Percentage (%) for W-BrC at Different Stages of UV Aqueous Photolysis

	oligomer (*m*/*z* > 215) to monomer (*m*/*z* < 215) ratio			BrC chromophore percentage (%) from DBE vs C		
sample	0 min	mid-point	120 min	0 min	mid-point	120 min
Piedmont wildfire	17.9	22.4	25.4	30.1	32.8	31.2
Piedmont prescribed	20.4	22.8	18.8	30.3	35.7	36.5
Coastal Plain wildfire	16.8	17.4	18.7	30.0	35.7	34.3
Coastal Plain prescribed	20.5	22.1	22.8	26.0	29.1	31.5
Blue Ridge wildfire	15.1	26.0	29.5	31.1	39.8	40.2
Blue Ridge prescribed	19.1	27.3	29.1	30.5	33.1	32.0

#### 3.2.3 Identifying Peaks in the Mass Spectra Corresponding to Potential Chromophores

It is difficult to determine conclusively which peaks in a mass spectrum correspond to chromophores and are therefore responsible for trends observed in the UV-vis absorption spectra, especially without employing some method of separation before the mass spectrometer, such as high-performance liquid chromatography equipped with a photodiode array detector (HPLC-PDA).^36−38^ However, Lin et al. introduced a method that makes it possible to identify potential chromophores by plotting the double bond equivalents (DBE; eq 1), calculated from the number of carbon and nitrogen atoms in an assignment, versus the number of carbon atoms (C).^38^ With this method, a larger DBE/C ratio implies a larger degree of conjugation with a higher probability of an uninterrupted conjugation structure that can potentially lead to the absorption of visible light. Thus, peaks that might be considered as part of BrC can be identified in regions of a high DBE/C ratio. This approach has been used in various UHR-MS analyses of atmospheric BrC samples.^17,38−40^

**Figure 8:**
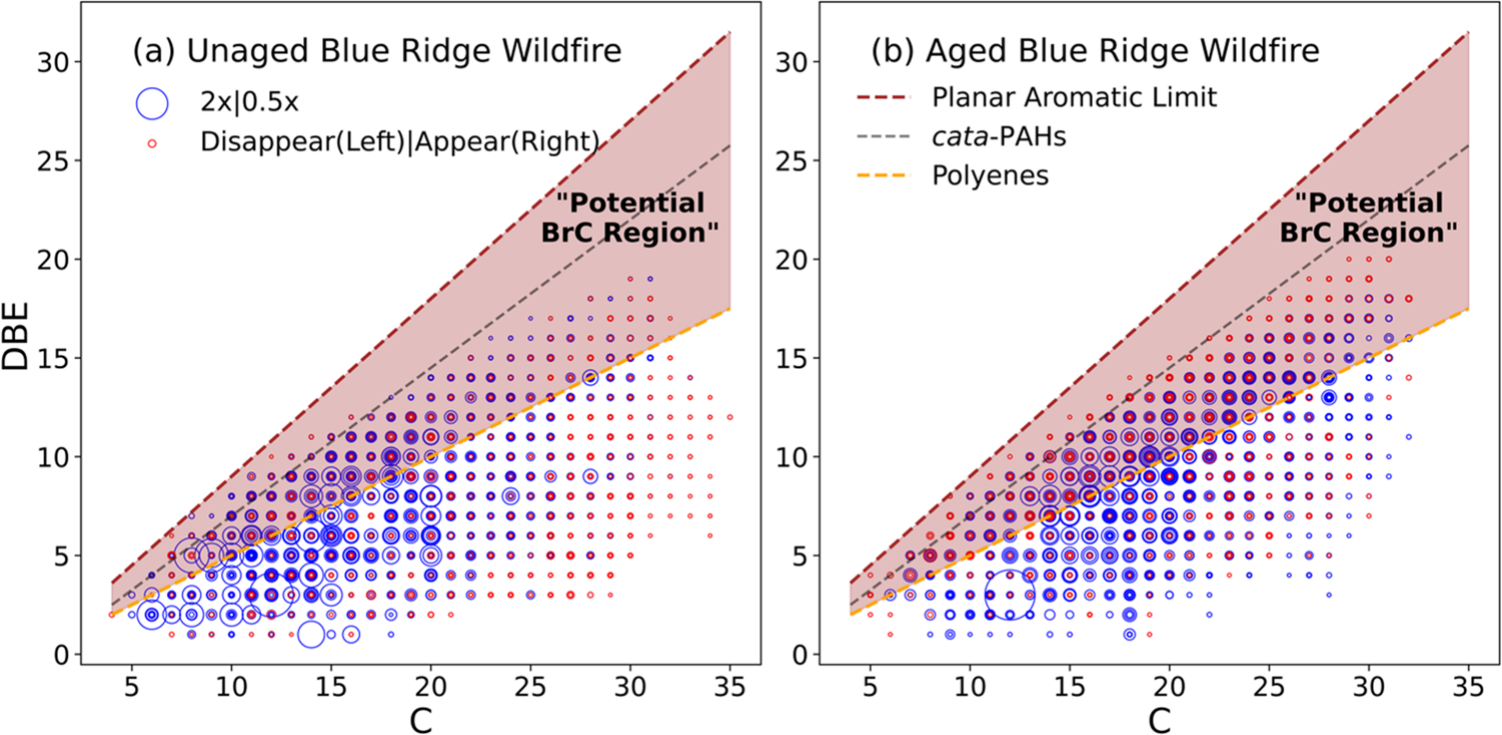
Double bond equivalent (DBE) value vs number of carbon (C) of assigned ESI-UHR-MS peaks in a Blue Ridge wildfire W-BrC sample: (a) before aqueous photolysis and (b) after a 2 h UV photolysis. Only assigned species that are unique in each spectrum (in red) or have significant change in abundance (>2× or <0.5×, in blue) are plotted here to simplify the plot. Individual marker size is proportional to intensity of each species. DBE vs C lines of reference materials of polyenes (C_*x*_H_*x*+2_), *cata*-PAHs^38^ and theoretical DBE limit for maximally condensed PAHs (“Planar Aromatic Limit”)^41^ are also plotted. The area between polyenes and the planar aromatic PAHs limit is shaded and marked as the “potential BrC region” to identify potential chromophores.

In Figure 8, we show the DBE vs C plot of water-soluble particulate matter from the combustion of a Blue Ridge sample under wildfire conditions, both pre-aqueous photolysis (Figure 8a) and post-aqueous photolysis (Figure 8b).^16,33−35^ To better visualize the change with photolysis, only species that demonstrate significant change in intensity (disappear/appear after photolysis or double/decrease by at least a factor of two) are plotted. The DBE vs C lines of three reference species are also shown: conjugated polyenes (C_*x*_H_*x*+2_, DBE = 0.5 × C; orange line), *cata*-PAHs (DBE = 0.75 × C – 0.5; black line)^38^ and the theoretical DBE limit for maximally condensed PAHs determined from fossil hydrocarbons (DBE = 0.9 × C; “planar aromatic limit”; red line).^41^ Species possessing a DBE/C ratio exceeding the conjugated polyene reference (0.5 × C) can potentially contribute to absorption in the visible range as BrC chromophores due to their uninterrupted conjugation structure.^38^ As a species’ DBE/C ratio increases closer to the polyene, *cata*-PAHs and maximally condensed PAHs DBE limit reference lines, respectively, the level of inferred conjugation increases so individual species can potentially absorb light more strongly.^42^ In Figure 8, we label the region between the polyene and maximally condensed PAHs DBE limit reference lines as the “potential BrC region”, indicating the potential of peaks appearing here to be visible chromophores.

We see a clear decrease in the intensity of smaller C_6–13_ species and the formation of larger species near the *cata*-PAHs reference in the C_>20_ range. In addition, the density of signals inside the “potential BrC region” increases. To better quantify this shift of density into the BrC region, a “BrC percentage” was calculated by normalizing the sum of signal intensity for peaks appearing within the BrC region by the total signal intensity of all plotted peaks. These calculated BrC percentages for all samples are listed in Table 1. These percentages are 26–31% before photolysis, increase to 29–40% at the midpoint of photolysis, and finally end up at 31–40% at the conclusion of the 2 h photolysis period. The majority of the observed increase in the BrC percentage happens by the mid-point, i.e., the time at which the visible photo-enhancement peaks, just as was observed for the oligomer-to-monomer ratio (also shown in Table 1). Taken together, these observations suggest that the aqueous photolysis process is potentially producing BrC chromophores that have a larger molecular weight and a larger degree of unsaturation.

Hopstock and co-workers explored the impacts of condensed phase photolysis on primary aerosols collected from the burning of urban materials using a similar DBE vs C analysis.^17^ They observed a similar trend for photo-enhanced samples in which smaller potential BrC chromophore (C_6–9_) signals were depleted, and larger potential BrC chromophore (C_16–20_) signals were enhanced.^17^

A caveat to note with our analysis is that peak abundances in Fourier transform–ion cyclotron (FT-ICR) mass spectra do not relate linearly to the concentration of corresponding species due to issues such as differing ionization efficiencies among species and matrix effects.^43^ Therefore, intensity-weighted metrics such as the oligomer-to-monomer ratio and the BrC percentage derived from FT-ICR mass spectrometry serve merely to demonstrate trends and cannot be used for quantification. In addition to the nonlinearity of the FT-ICR response, our approach is also biased toward species that have high ionization efficiency under negative ion ESI, so species such as PAHs that cannot be ionized efficiently will not be accounted for in this assessment.

### 3.3 Atmospheric Implications

The results of this work have four main implications:

1) Across all types of fuels and combustion conditions (wildfire and prescribed) examined in this work, aqueous photolysis consistently affected the absorption spectrum of biomass burning water-soluble brown carbon (W-BrC). Photobleaching at UV wavelengths and photo-enhancement at visible wavelengths was observed for all samples with photobleaching being the more dominant effect. The shapes of the absorption spectra were consequently affected leading to decreased absorption Ångström exponent (AAE) values.
2) The effect of prescribed-fire and wildfire conditions on biomass burning W-BrC production varies with the fuel bed composition. For fuel beds without duff (Piedmont and Coastal Plain), prescribed burning produces W-BrC with a slightly higher AAE than under wildfire conditions. This observation is attributed to the increased moisture content in the fuel under prescribed conditions, as wetter fuels lead to less complete combustion and result in smoldering.^44^ Conversely, for the Blue Ridge sample, which is characterized by a substantial duff layer, wildfire conditions lead to a higher AAE for W-BrC, which we attribute to the smoldering of the duff layer that does not occur under prescribed conditions.^45^ While this work analyzes only the water-soluble portion, the findings highlight the importance of considering both fuel composition and burn conditions when assessing the potential climate impact of aerosols produced from biomass burning, in general.
3) Due to the changes in the spectral shape caused by the photobleaching in the UV region, W-BrC spectra from combustion of fuel beds from the different ecoregions and under both wildfire and prescribed burn conditions appear very similar after exposure to UV light with AAE values of approximately 5. This observation suggests that biomass burning W-BrC from a variety of sources and produced under a variety of conditions may exhibit more similar absorption spectra after as little as 5 h in the atmosphere, irrespective of their initial differences.
4) Aqueous photolysis of the water-soluble fraction of biomass burning aerosols can potentially increase the extent of oligomerization with some of the oligomers formed acting as chromophores.

There are a few caveats to these conclusions that warrant mentioning. First, the aqueous photolysis of BrC investigated herein was confined to water-soluble fractions. It is important to acknowledge that the photolysis processes in atmospheric particles, particularly those occurring at lower concentrations, such as in fog or cloud droplets,^23^ may differ significantly from our findings. Second, the photolytic mechanisms observed may include not only direct photolysis but also photoinitiated reactions, such as hydroxyl radical (OH) oxidation, which were not distinctly differentiated in this work.^16^ Lastly, the scope of our research was geographically limited to biomass burning fuel beds sourced from the state of Georgia. Consequently, the W-BrC derived from the combustion of fuel beds from other regions could exhibit different chemical compositions and photobleaching/photo-enhancement behavior. This possibility underscores the need for continued research into BrC photolysis using biomass fuel beds with diverse geographical origins.

## Supporting Information Available

Organic carbon concentration of each W-BrC sample; Light source information; UV-vis spectra progression of all samples; Photo-enhancement effect in this work compared to literature; Levoglucosan spiking experiment; ESI-UHR-MS spectra of all samples; List of possible monoaromatic species detected; Details of use of ChatGPT for text editing and refinement (PDF)
