# Supplementary material for: Aqueous Photolysis of Water-Soluble Brown Carbon from Simulated Prescribed and Wildfire Biomass Burning
Source: ACS EST Air. 2024 Jul 10;1(9):989–99. doi: 10.1021/acsestair.4c00016 (PMC11406525; doi:10.1021/acsestair.4c00016)
Supplement: Supplementary file 1 — ea4c00016_si_001.pdf [file ea4c00016_si_001.pdf]

Supporting Information

Aqueous photolysis of water-soluble brown carbon from simulated prescribed and wildfire biomass burning

*Mingrui Sun[a], Chase K. Glenn[b†], Omar El Hajj[b‡], Kruthika V. Kumar[b], Anita Anosike[b], Robert Penland[b], Mac A. Callaham, Jr.[c], E. Louise Loudermilk[c], Joseph J. O'Brien[c], Rawad Saleh[b], Geoffrey D. Smith[a]**

[a] Department of Chemistry, University of Georgia, Athens, Georgia, 30602, United States

[b] School of Environmental, Civil, Agricultural and Mechanical Engineering, University of Georgia, Athens, Georgia, 30602, United States

[c] U.S. Department of Agriculture Forest Service, Southern Research Station, Athens Prescribed Fire Science Laboratory, Athens, Georgia, 30602, United States

[†] Now at Aerodyne Research, Billerica, Massachusetts, 01821, United States

[‡] Now at Tofwerk USA, Boulder, Colorado, 80301, United States

* Email: geosmith@uga.edu, Phone: +1 706-583-0478

Table of Contents

Table S1. Organic Carbon Concentration of Each W-BrC Sample in This Work 3

Table S2. Integrated photon flux in 290-340 nm region for solar spectrum and UV lamp 3

Figure S1. Spectrum of UV lamp compared to Athens, GA's 07/15/2023 24-hour average solar spectrum 4

Figure S2. Demonstration of Spectroradiometer Measurement in the Photoreactor 5

Figure S3. UV-Vis spectrum of azoxybenzene actinometer experiment...................................6

Figure S4. Beaker (Borosilicate Glass) Filtering Experiment on Blue Ridge Wildfire W-BrC........7

Figure S5. Comparison of UV Lamp Spectrum to with Beaker Filter (borosilicate glass) Spectrum..7

Figure S6. UV-vis progression of Blue Ridge Wildfire sample with (right) and without (left) beaker (borosilicate glass) filtering..8

Table S3. Photo-enhancement effect observed in previous studies and the present work.........9

Figure S7. Progression of normalized absorption at wavelengths of (a) 365 nm and (b) 400 nm during aqueous UV photolysis. 9

Figure S8. UHR-MS spectra of Blue Ridge wildfire condition W-BrC sample...........................10

Figure S9. UHR-MS spectra of Blue Ridge prescribed condition W-BrC sample.......................11

Figure S10. UHR-MS spectra of Piedmont wildfire condition W-BrC sample...........................12

Figure S11. UHR-MS spectra of Piedmont prescribed condition W-BrC sample. 13

Figure S12. UHR-MS spectra of Coastal Plain wildfire condition W-BrC sample. 14

Figure S13. UHR-MS spectra of Coastal Plain prescribed condition W-BrC sample..................15

Levoglucosan spiking experiment 16

Figure S14. Levoglucosan standard spiking experiment in water and G-Wise W-BrC matrix...16

Table S4. Possible monoaromatic species detected in W-BrC sample from each sample.........16

Use of ChatGPT for text editing and refinement..19

References:**...64**

Table S1. Organic Carbon Concentration of Each W-BrC Sample in This Work

**Burn Date***	**Experiment**	**OC Concentration of Aqueous Extract (ug/mL)**
**10/28/22**	P-Prescribed	49.95 ± 4.05
**11/1/22**	P-Wild	29.70 ± 3.00
**11/2/22**	CP-Wild	34.35 ± 3.15
**11/6/22**	CP-Prescribed	51.75 ± 4.05
**11/9/22**	BR-Wild	72.00 ± 5.10
11/14/22	BR-Wild	81.15 ± 5.55
**11/15/22**	BR-Prescribed	50.25 ± 4.05

***:Date in bold text are samples that were examined in this work.**

Table S2. Integrated photon flux in 290-340 nm region for solar spectrum and UV lamp

Integrated Flux of UV Lamp (Photons/cm^2/nm/s)	Integrated Flux of 24H Avg Solar Spectrum (Photons/cm^2/nm/s) *	Integrated Flux of Noon Solar Spectrum (Photons/cm^2/nm/s) *	Equivalent Solar Exposure Time Ratio (24H-Avg)**	Equivalent Solar Exposure Time Ratio (Noon)**

$2.86*10^{15}$	$3.29*10^{14}$	$9.72*10^{14}$	2.5	0.9

*: "Quick TUV" calculator. (https://www.acom.ucar.edu/Models/TUV/Interactive_TUV/)[1]

**: The equivalent time ratio here is calculated by taking the ratio of lamp flux to solar flux at 290 – 340 nm range where the UV lamp emission resided.

Figure S1. Spectrum of UV lamp compared to Athens, GA's 07/15/2023 24-hour average solar spectrum obtained from "Quick TUV" calculator. (https://www.acom.ucar.edu/Models/TUV/Interactive_TUV/)[1]The UV lamp spectrum shape was obtained with a spectroradiometer (RPS-900, International Light Technologies) and then scaled by the photon flux as measured using the azoxybenzene chemical actinometer.[2] Details about the spectral measurement are shown in Figure S2.

The following parameters were used to generate the solar spectrum using the Quick TUV calculator:

-Latitude/Longitude: 33.95˚N 83.357˚W

-Date and Time: July 15, 2023, 4:00:00 am - July 16, 2023, 4:00:00 am GMT

-Overhead Ozone: 300 du

-Surface Albedo: 0.1

-Ground Altitude: 0 km

-Measured Altitude: 0 km

-Clouds Optical Depth/Base/Top: 0:00/4.00/5.00

-Aerosols Optical Depth/S-S Albedo/Alpha: 0.235/0.990/1.000

-Sunlight Direct Beam/Diffuse Down/Diffuse Up: 1.0/1.0/0.0

-4 Streams Transfer Model

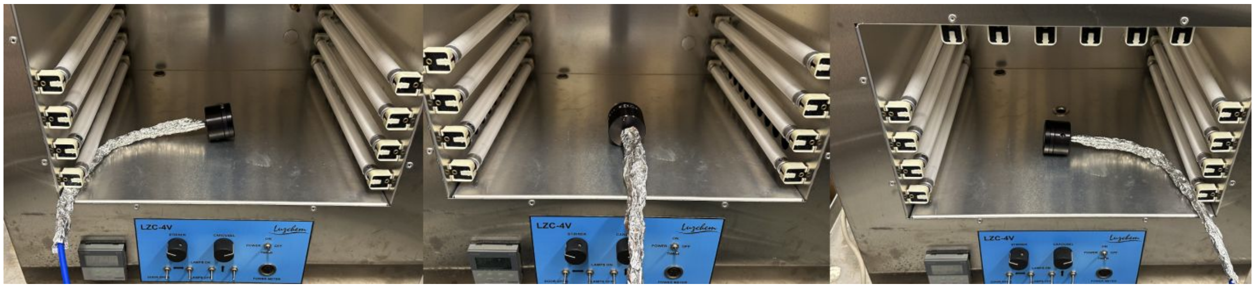

Figure S2. Demonstration of Spectroradiometer Measurement in the Photoreactor. The emission spectrum inside the UV reactor was measured with a spectroradiometer equipped with a cosine diffusor facing to the left, center and right of the chamber. The overall emission spectrum experienced by the cuvette was the weighted average of these three measurements ((center*2 + left + right)*0.25). Sensor head pointing up wasn't measured as the cuvette have a plastic stopper which would block direct emission from the top. Finally, the spectrum shape obtained was scaled using chemical actinometer measurement resulting in scaled spectrum shown in Figure S1.

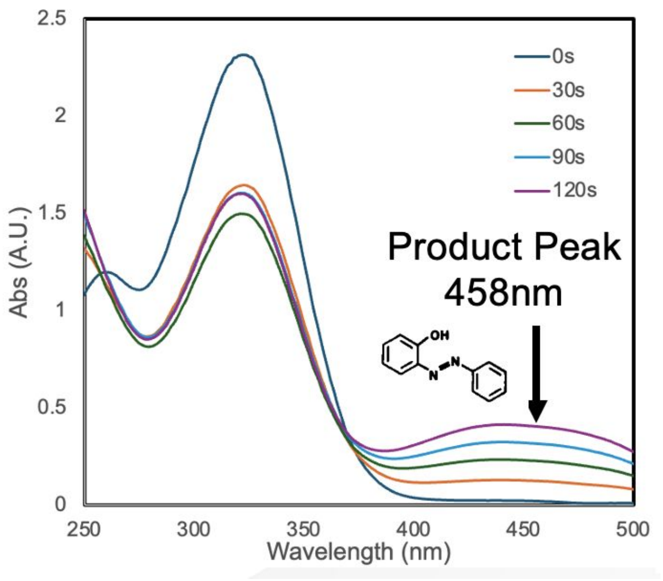

Figure S3. UV-Vis spectrum of azoxybenzene actinometer experiment. Photo-isomerization product peak at 458 nm with known molar extinction coefficient is highlighted.

The azoxybenzene actinometer experiment was conducted using an azoxybenzene/ethanol solution with 0.2 mM azoxybenzene and 0.6 mM KOH. 3 mL of this solution was added to the cuvette placed in the center of the photo-reactor. During 2 min of UV exposure, the UV-Vis absorption spectrum of the solution was collected every 10 s. The azoxybenzene photo-isomerization product has a known molar extinction coefficient of 7600 L mol^{-1} cm^{-1},[3] which is used to convert the absorption at 458 nm to product concentration, P. The photon flux in the UV range is given by:[4]

$$A_0 \ln\left(1 - \frac{P}{A_0}\right) = -\phi_r I_0 t$$

in which A_0 is the initial azoxybenzene concentration, ϕ_r is the quantum yield of this reaction (equal to 0.21 in the UV range),[4] t is the duration of the exposure and I_0 is the incident light intensity. This equation is linear up to 40% conversion to the product.[4]

The incident light intensity, I_0, was converted to photon flux, F_0 (in units of photon/cm^2/s) with the following equation:

$$\mathrm{F}_0 = I_0 * V * \frac{N_A}{\mathrm{Area}}$$

in which V is the volume of solution (3 mL), N_A is Avogadro's constant, and Area is the area of solution in cuvette being exposed to light. In our case, Area is taken to be the area of the four sides of the cuvette exposed to the light, which equals 12 cm^2.

Overall, the photon flux inside the chamber was determined to be 3.72 * 10^{15} photons/cm^2/s. In the borosilicate beaker filtering experiment this flux was 2.78 * 10^{15} photons/cm^2/s. These values were then used to scale the spectrum obtained with the spectroradiometer to photon flux units in Figure S1 and Figure S5.

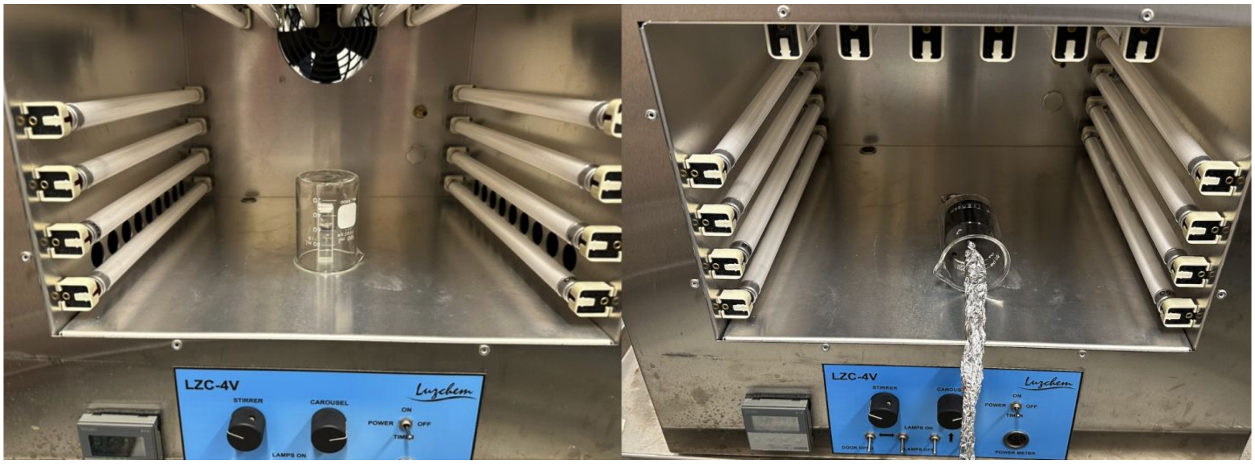

Figure S4. Beaker (Borosilicate Glass) Filtering Experiment on Blue Ridge Wildfire W-BrC. The emission spectrum shape from this setup was determined in similar manner as in Figure S2 but with the beaker put on top of the cosine sensor head.

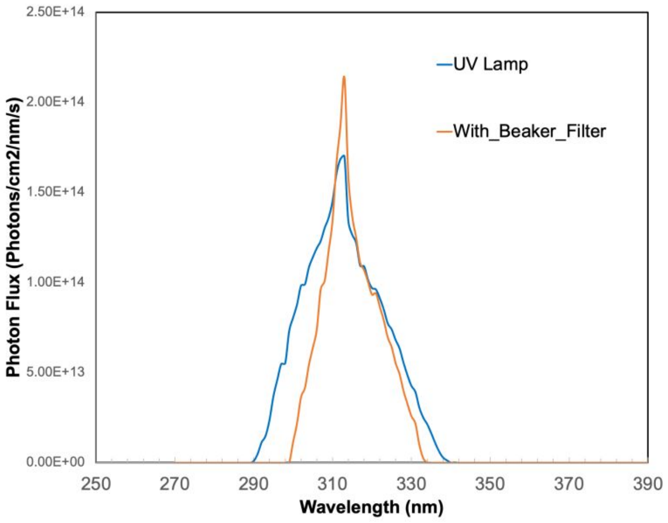

Figure S5. Comparison of UV Lamp spectrum to spectrum with beaker (borosilicate glass) as filter spectrum. The beaker efficiently removes the sub-300 nm emission from the lamp without altering the longer-wavelength part of the spectrum significantly.

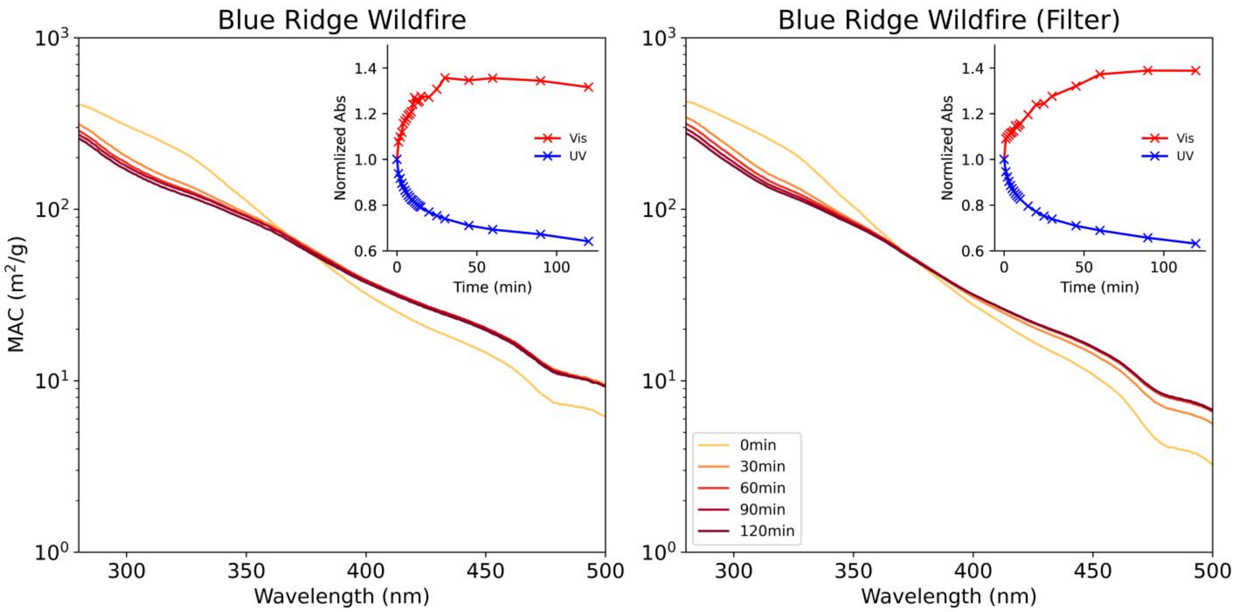

Figure S6. UV-vis progression of Blue Ridge Wildfire sample with (right) and without (left) beaker (borosilicate glass) filtering. The filtered data was obtained by utilizing two filters collected on 11/14/22 (Blue Ridge Wildfire) extracted following the same protocol as described in main text. The removal of sub-300 nm emission has no noticeable impact, as these two experiments show similar trends in terms of photo-enhancement and photo-bleaching (insets).

Table S3. Photo-enhancement effect observed in previous studies and the present work.

Reference	Fuel	Lamp Wavelength	Generation Temperature (°C)	Peak of $Abs_{365}[t]/Abs_{365}[t_0]$	Peak of $Abs_{400}[t]/Abs_{400}[t_0]$
Wong et al.[5]	Cherry wood	UVB	215	1.68	2.57
Hems et al.[6]	Red Oak/Pine	UVB	400		2.50
Choudhary et al.[7]	Lodge Pole Pine	UVB	500	1.39	3.35
This work	Piedmont, Coastal Plain or Blue Ridge eco-regions	UVA/B	300 - 460*	1.38-1.05	1.20-1.05

*: This temperature range was recorded with the overhead FTIR camera on fuel bed during the flaming phase of the burning.

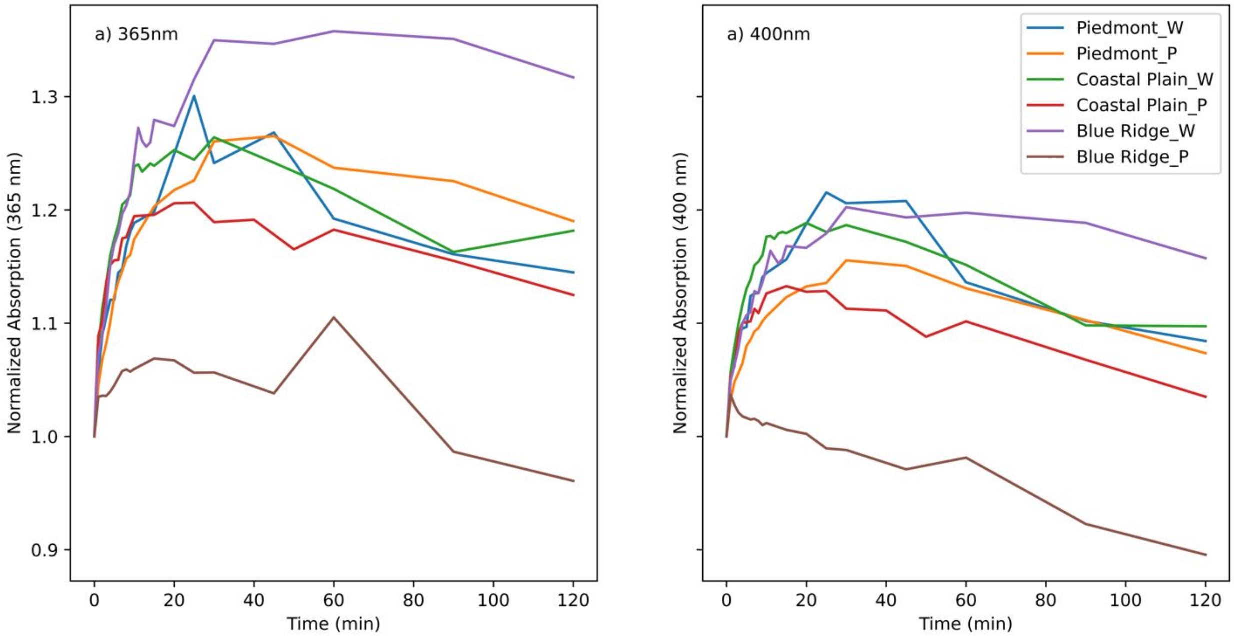

Figure S7. Progression of normalized absorption at wavelengths of (a) 365 nm and (b) 400 nm during aqueous UV photolysis. All absorption values shown here were normalized to 0 min absorption values at 365 nm or 400 nm.

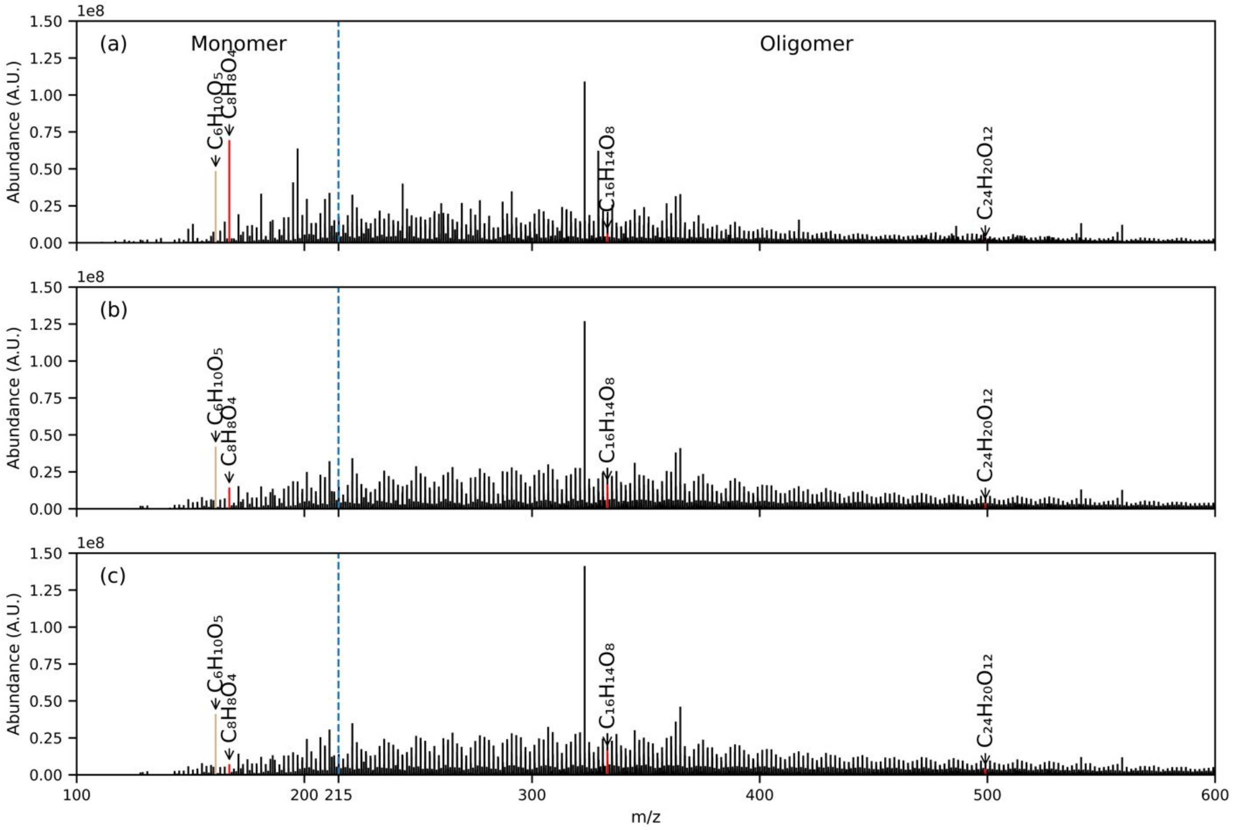

Figure S8. UHR-MS spectra of Blue Ridge wildfire condition W-BrC sample. (a), (b), (c) corresponding to before, midpoint of (60 minutes), and end of 2-hour photolysis. Assigned peaks, $C_8H_8O_4$ (vanillic acid), $C_{16}H_{14}O_8$ (vanillic acid dimer) and $C_{24}H_{20}O_{12}$ (vanillic acid trimer), are highlighted (in red) here as example for oligomer production during aqueous photolysis. Assigned peak $C_6H_{10}O_5$ (levoglucosan, in tan) is also highlighted as a common biomass burning tracer.

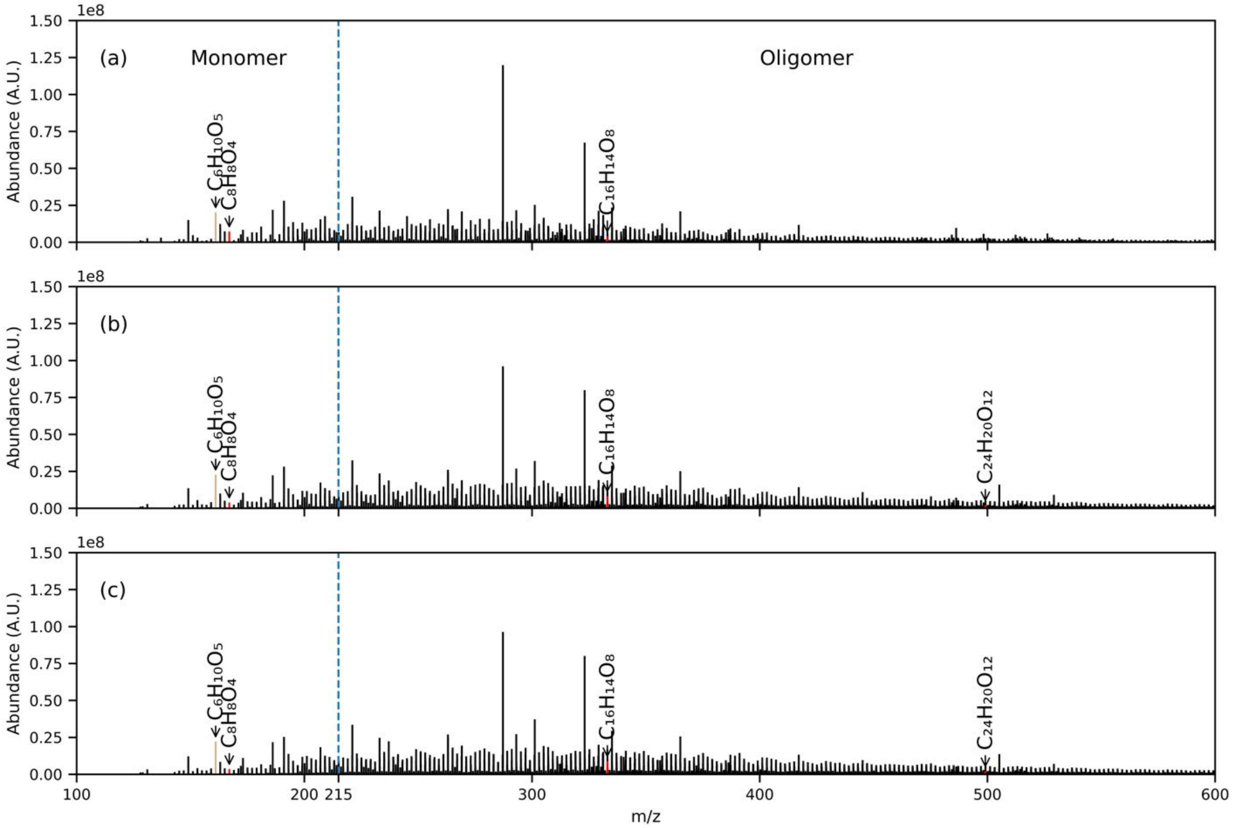

Figure S9. UHR-MS spectra of Blue Ridge prescribed condition W-BrC sample. (a), (b), (c) corresponding to before, midpoint of (60 minutes), and end of 2-hour photolysis. Assigned peaks, $C_8H_8O_4$ (vanillic acid), $C_{16}H_{14}O_8$ (vanillic acid dimer) and $C_{24}H_{20}O_{12}$ (vanillic acid trimer), are highlighted (in red) here as example for oligomer production during aqueous photolysis. Assigned peak $C_6H_{10}O_5$ (levoglucosan, in tan) is also highlighted as a common biomass burning tracer.

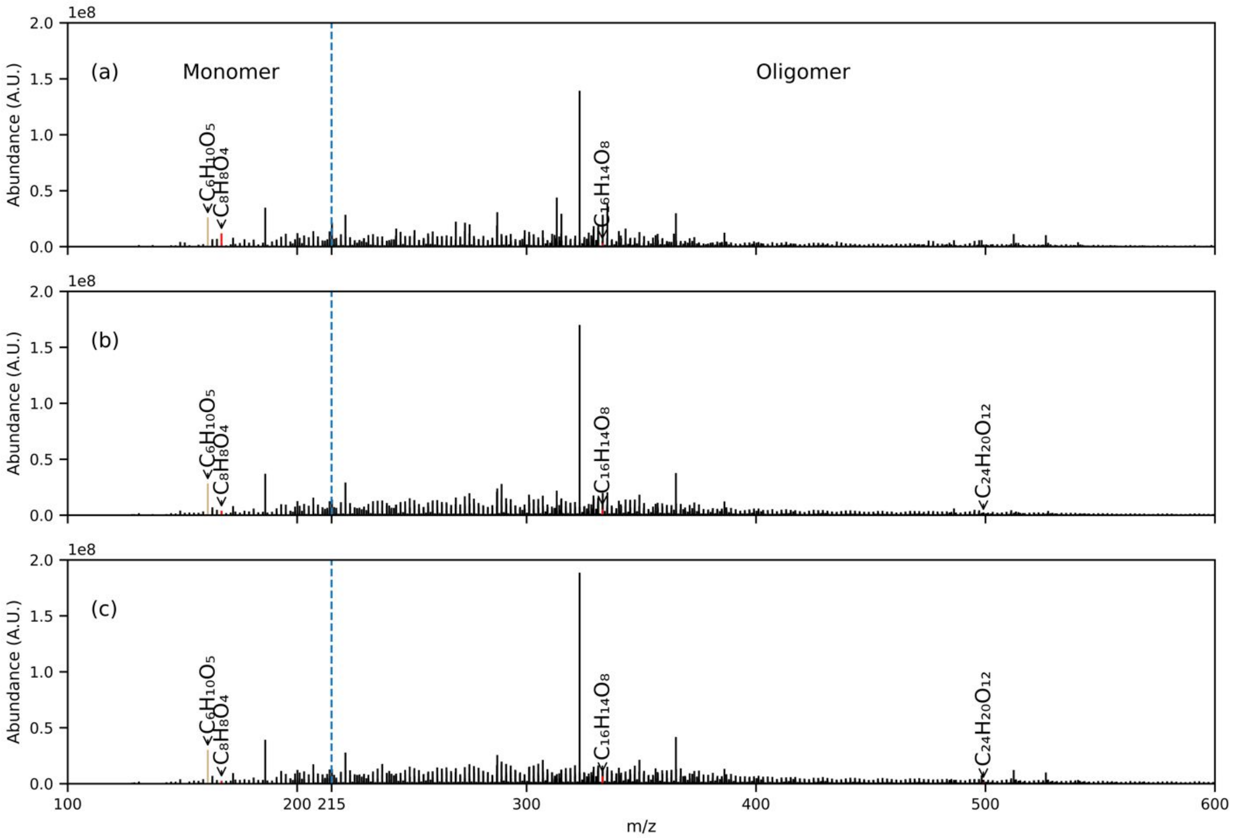

Figure S10. UHR-MS spectra of Piedmont wildfire condition W-BrC sample. (a), (b), (c) corresponding to before, midpoint of (30 minutes), and end of 2-hour photolysis. Assigned peaks, $C_8H_8O_4$ (vanillic acid), $C_{16}H_{14}O_8$ (vanillic acid dimer) and $C_{24}H_{20}O_{12}$ (vanillic acid trimer), are highlighted (in red) here as example for oligomer production during aqueous photolysis. Assigned peak $C_6H_{10}O_5$ (levoglucosan, in tan) is also highlighted as a common biomass burning tracer.

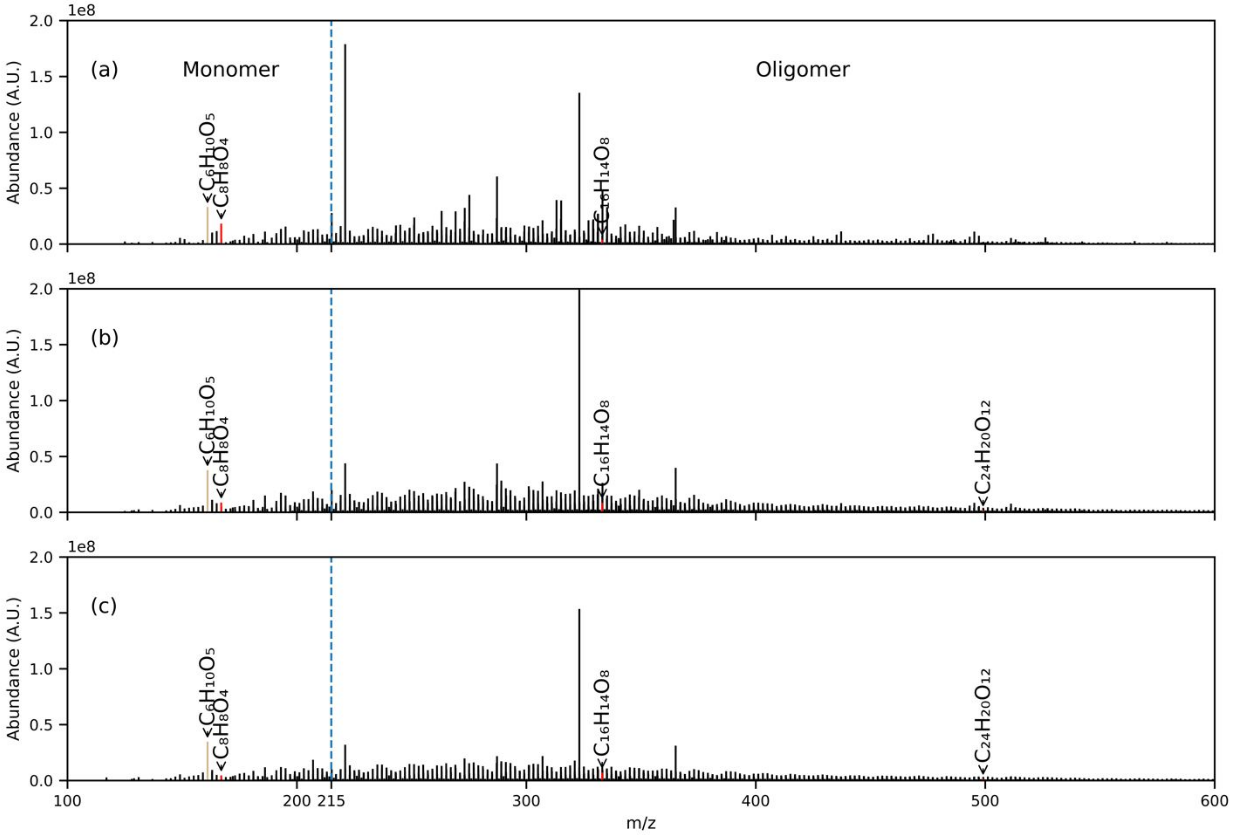

Figure S11. UHR-MS spectra of Piedmont prescribed condition W-BrC sample. (a), (b), (c) corresponding to before, midpoint of (45 minutes), and end of 2-hour photolysis. Assigned peaks, $C_8H_8O_4$ (vanillic acid), $C_{16}H_{14}O_8$ (vanillic acid dimer) and $C_{24}H_{20}O_{12}$ (vanillic acid trimer), are highlighted (in red) here as example for oligomer production during aqueous photolysis. Assigned peak $C_6H_{10}O_5$ (levoglucosan, in tan) is also highlighted as a common biomass burning tracer.

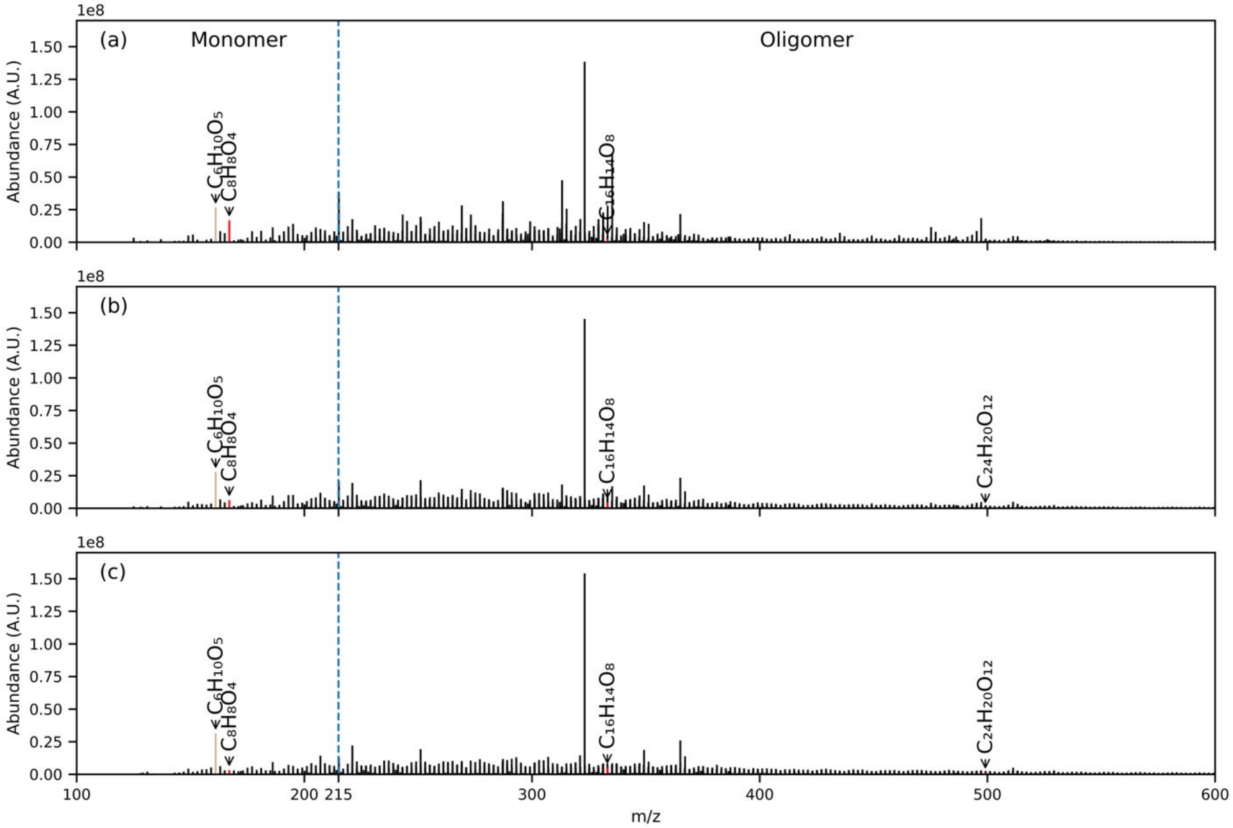

Figure S12. UHR-MS spectra of Coastal Plain wildfire condition W-BrC sample. (a), (b), (c) corresponding to before, midpoint of (30 minutes), and end of 2-hour photolysis. Assigned peaks, $C_8H_8O_4$ (vanillic acid), $C_{16}H_{14}O_8$ (vanillic acid dimer) and $C_{24}H_{20}O_{12}$ (vanillic acid trimer), are highlighted (in red) here as example for oligomer production during aqueous photolysis. Assigned peak $C_6H_{10}O_5$ (levoglucosan, in tan) is also highlighted as a common biomass burning tracer.

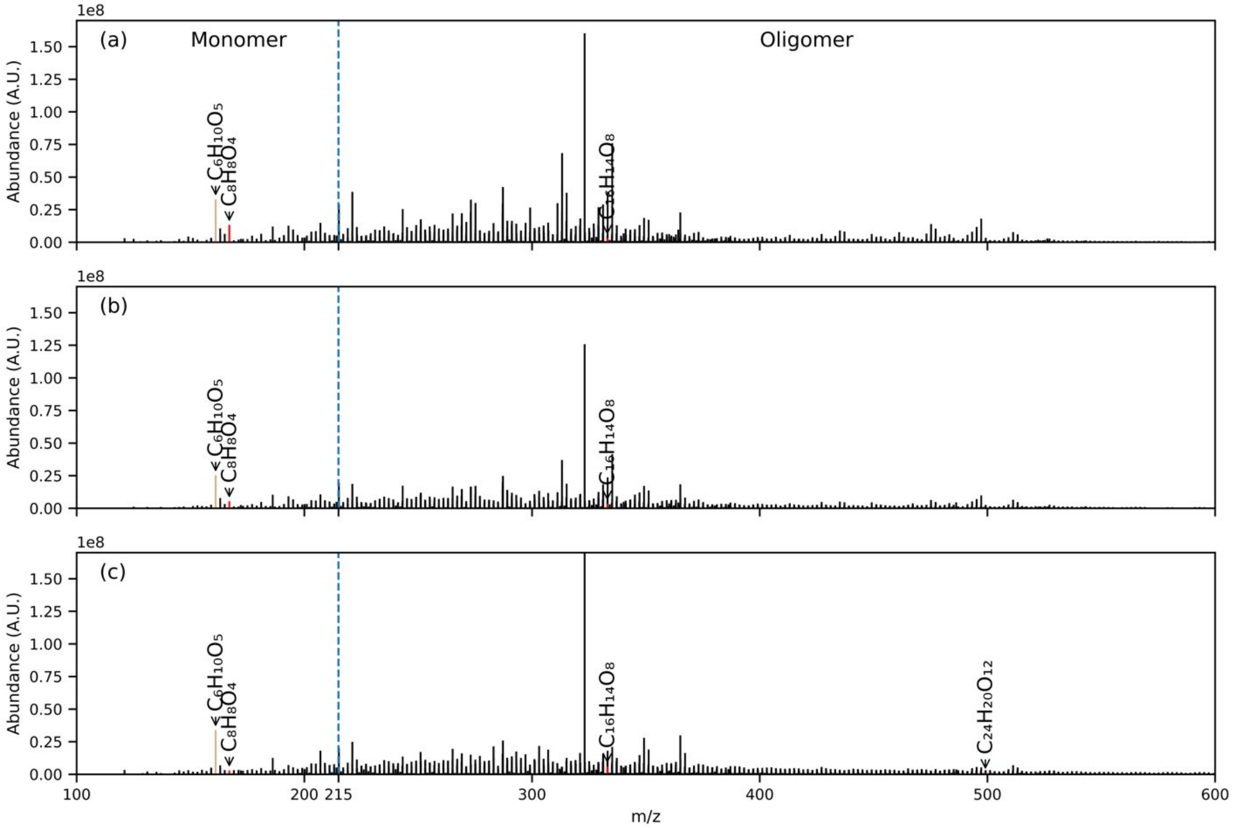

Figure S13. UHR-MS spectra of Coastal Plain prescribed condition W-BrC sample. (a), (b), (c) corresponding to before, midpoint of (20 minutes), and end of 2-hour photolysis. Assigned peaks, $C_8H_8O_4$ (vanillic acid), $C_{16}H_{14}O_8$ (vanillic acid dimer) and $C_{24}H_{20}O_{12}$ (vanillic acid trimer), are highlighted (in red) here as example for oligomer production during aqueous photolysis. Assigned peak $C_6H_{10}O_5$ (levoglucosan, in tan) is also highlighted as a common biomass burning tracer.

We performed a levoglucosan standard spiking experiment to address concerns about ion cluster issue of dimers assigned in ESI(-)-UHR-MS analysis of this work. In this experiment, we added levoglucosan standard to an aqueous Blue Ridge (wildfire) sample at various concentrations. We determined that the dimer/monomer ratio did not increase with addition of levoglucosan except at the highest concentration (1000 µg/mL – see Figure S14). Given that the concentration of the entire organic carbon fraction in the samples ranged from 30 µg/mL to 70 µg/mL, we conclude that ion clustering was not likely to contribute significantly to the observations of dimers and trimers (Figures 6 and 7) and, instead, they are more likely to have formed in the particles.

Note that we did not have vanillic acid, so we could not do a similar doping experiment. But, we expect that it would also only show enhanced dimer formation at concentrations much larger than could be found in the samples.

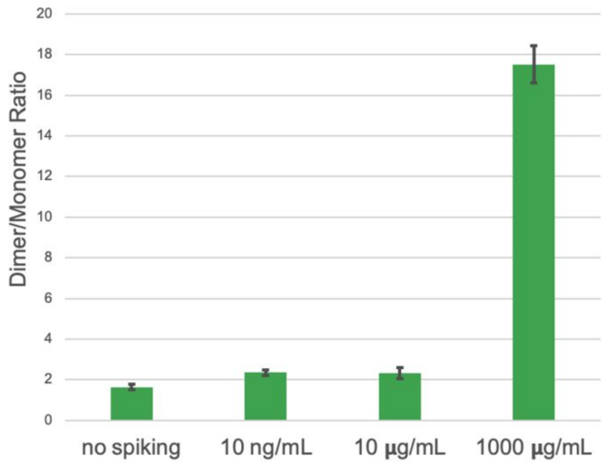

Figure S14. Levoglucosan standard spiking experiment in aqueous extract of Blue Ridge Wildfire aerosol sample.

Table S4. Possible monoaromatic species detected in W-BrC sample from each sample. P- Piedmont, CP- Coastal Plain, BR- Blue Ridge. Y/N here indicates whether a species is present in the assigned mass spectrum or not.

Formula	Calculated Neutral Mass	Possible Identity	P(W)	P(P)	CP(W)	CP(P)	BR(W)	BR(P)
$C_7H_6O_2$	122.0367845	Salicylaldehyde	N	N	N	Y	Y	N
$C_7H_8O_2$	124.0524195	Guaiacol	N	N	N	N	Y	N
$C_8H_8O_2$	136.052421	Methyoxybenzaldehyde or 2-Methylbenzoic acid	N	N	N	Y	Y	N
$C_7H_6O_3$	138.0316566	3-hydroxybenzoic acid	Y	Y	Y	Y	Y	Y
$C_8H_8O_3$	152.0473387	Syringol	Y	Y	Y	Y	Y	Y
$C_7H_7NO_3$	153.042576	Methoxy/Methyl derivate of Nitrophenol	N	N	N	N	Y	Y
$C_9H_8O_3$	164.0473415	p-Coumaric acid	Y	Y	Y	Y	Y	Y
$C_8H_6O_4$	166.0266362	Phthalic acid	Y	Y	Y	Y	Y	Y
$C_8H_7NO_3$	165.0426078		N	N	N	N	Y	N
$C_9H_{10}O_3$	166.062997	Veratraldehyde or Acetovanillone	Y	Y	Y	Y	Y	Y
$C_8H_8O_4$	168.0422594	Vanillic acid	Y	Y	Y	Y	Y	Y
$C_7H_7NO_4$	169.0374906	4-Nitroguaiacol	N	N	N	N	Y	N
$C_{10}H_{10}O_3$	178.0629936	Coniferaldehyde	Y	Y	Y	Y	Y	Y
$C_9H_{10}O_4$	182.0579255	Homovanillic acid	Y	Y	Y	Y	Y	Y
$C_8H_9NO_4$	183.0531277	Nitrophenol	N	N	N	N	Y	N
$C_{12}H_{12}O_3$	204.0786374	Dimethyl-methoxy-coumarin	Y	Y	Y	Y	Y	Y
$C_{11}H_{12}O_4$	208.0735803	Sinapaldehyde	Y	Y	Y	Y	Y	Y

Table S4 continued. Possible Monoaromatic Species and Their Corresponding Dimer, Trimer Signal Abundance Changed After Photolysis.

Monomer	Normalized Abundance (120 min/0 min)						Dimer	Normalized Abundance (120 min/0 min)						Trimer	Normalized Abundance (120 min/0 min)					
	P(W)	P(P)	CP(W)	CP(W)	BR(W)	BR(P)		P(W)	P(P)	CP(W)	CP(P)	BR(W)	BR(P)		P(W)	P(P)	CP(W)	P(P)	BR(W)	BR(P)
$C_7H_6O_2$	N*	N	N	1.1	0	N	$C_{14}H_{10}O_4$	N	N	N	0.94	N	N	$C_{21}H_{14}O_6$	N	N	N	1.8	N	N
$C_7H_8O_2$	N	N	N	N	0	N	$C_{14}H_{14}O_4$	N	N	N	N	0.5	N	$C_{21}H_{20}O_6$	N	N	N	N	1.0	N
$C_8H_8O_2$	N	N	N	1.7	0	N	$C_{16}H_{14}O_4$	N	N	N	0.8	0.7	N	$C_{24}H_{20}O_6$	N	N	N	+inf**	0	N
$C_7H_6O_3$	0	0.7	0	0.6	0	0	$C_{14}H_{10}O_6$	1.7	1.8	1.7	2.2	2.8	2.5	$C_{21}H_{14}O_9$	+inf	+inf	+inf	+inf	+inf	2.3
$C_8H_8O_3$	0	0.6	0.3	0.6	0.3	0.4	$C_{16}H_{14}O_6$	1.9	1.3	1.1	1.5	1.6	1.6	$C_{24}H_{20}O_9$	1.7	1.3	1.6	1.6	2.1	2.1
$C_7H_7NO_3$	N	N	N	N	0.7	0	$C_{14}H_{12}N_2O_6$	N	N	N	N	+inf	N	$C_{21}H_{17}N_4O_{12}$	N	N	N	N	N	N
$C_9H_8O_3$	0.7	0.8	0.4	0.6	0.6	0.7	$C_{18}H_{14}O_6$	1.3	0.9	0.9	1.3	1.3	1.3	$C_{27}H_{20}O_9$	+inf	+inf	N	N	N	1.7
$C_8H_6O_4$	1.8	2.4	1.3	1.5	1.7	1.8	$C_{16}H_{10}O_8$	+inf	1.7	+inf	+inf	+inf	+inf	$C_{24}H_{14}O_{12}$	N	N	N	N	N	N
$C_8H_7NO_3$	N	N	N	N	0.9	N	$C_{16}H_{12}N_2O_6$	N	N	N	N	N	N	$C_{24}H_{17}N_3O_9$	N	N	N	N	N	N
$C_9H_{10}O_3$	0.5	0.4	0.4	0.5	0.4	0.6	$C_{18}H_{18}O_6$	1.2	0.7	0.7	0.9	1.0	1.1	$C_{27}H_{26}O_9$	1.0	0.9	1.3	1.3	1.4	1.6
$C_8H_8O_4$	0.23	0.25	0.2	0.23	0.10	0.5	$C_{16}H_{14}O_8$	2.4	1.8	2.0	1.9	2.6	2.3	$C_{24}H_{20}O_{12}$	+inf	+inf	+inf	+inf	3.8	+inf
$C_7H_7NO_4$	N	N	N	N	1.04	N	$C_{14}H_{12}N_2O_8$	N	N	N	N	N	N	$C_{21}H_{17}N_4O_{12}$	N	N	N	N	N	N
$C_{10}H_{10}O_3$	0.5	0.5	0.3	0.5	0.5	0.6	$C_{20}H_{18}O_6$	0.9	0.6	0.6	0.8	1.0	1.1	$C_{30}H_{26}O_9$	1.4	N	0	+inf	N	1.2
$C_9H_{10}O_4$	0.9	1.0	0.6	0.7	0.4	0.6	$C_{18}H_{18}O_8$	2.4	1.7	1.8	1.9	1.3	2.1	$C_{27}H_{26}O_{12}$	2.8	1.8	2.2	2.8	2.3	2.9
$C_8H_9NO_4$	N	+inf	N	N	0.89	N	$C_{16}H_{16}N_2O_8$	N	N	N	N	+inf	N	$C_{24}H_{23}N_3O_{12}$	N	N	N	N	N	N
$C_{12}H_{12}O_3$	0.5	0.4	0.4	0.6	0.4	0.5	$C_{24}H_{22}O_6$	0.9	0.6	0	0.8	0.9	1.0	$C_{36}H_{32}O_9$	N	N	N	N	N	N
$C_{11}H_{12}O_4$	1.0	0.8	0.8	0.8	0.8	0.8	$C_{22}H_{22}O_8$	1.3	1.1	1.2	1.3	1.5	1.5	$C_{33}H_{32}O_{12}$	N	N	N	N	N	N

*:N here means the ratio don’t exist as the signal don’t shown up at 0 min or 120 min.

**: +inf means signal exist at 120 min but not 0 min

Use of ChatGPT for text editing and refinement

ChatGPT 4.0[6] was used for refining the writing and for editing purposes. Most of the use of ChatGPT was in the Introduction section, though it was also used to some degree in the Abstract, Methods and Results & Discussion sections. Included, below, is a transcript of its use including input text, prompts and responses. Not everything produced by ChatGPT was incorporated into the manuscript, and in all cases the output was reviewed and modified, as appropriate, by the authors.

S

You

You are a atmospheric chemist, revise this introduction for a research paper for me. :Atmospheric aerosols possess the ability to alter incoming solar radiation through direct scattering or absorption of light. Among various aerosol species, Brown Carbon (BrC) is notable for its robust absorption of solar radiation, particularly within the UV region (280-400 nm), with the absorption capability diminishing at longer wavelengths, rendering its characteristic brownish color.1 As a potent light absorbent, BrC has been estimated to be responsible for up to 24% of the aerosol warming effect and can influence tropospheric photochemistry by reducing the amount of solar UV radiation reaching the Earth's surface.2,3 Upon emission into the atmosphere, the optical and chemical properties of BrC can continue to evolve through aging processes in various media like gas-phase, cloud, or fog. The exact impacts of BrC on climate depend on the aging process it endured and its composition.4 A principal source of atmospheric BrC is biomass burning, which manifests in forms like open vegetation fires or human biofuel burning activity. It's estimated that biomass burning BrC accounts for a significant 85% of global primary organic aerosol production.5 The optical properties of Biomass Burning BrC exhibit a high degree of variability depending on the biomass burning fuel type and conditions. Currently, biomass burning BrC poses a source of uncertainty in climate models, attributed to the limited understanding of its sources, aging processes, and optical properties.6 Two factors that is understudy are prescribed/wildfire condition and duff burning. Prescribed burning is a forest management strategy aiming to mitigate the escalating economic and ecological risks posed by wildfires. From a combustion standpoint, a main

distinction between wildfire and prescribed fire lies in the amount of moisture content in the fuels; prescribed fires are conducted in high moisture content fuel conditions, especially following rain, as opposed to wildfires which occur in drier fuel conditions like during drought event. In southeastern US, most forest fire are prescribed fire as opposed to wildfire.7 Due to the prevalent of prescribed burning, it is important to assess its aerosol emission impact, which can help develop better fire management policy to reduce its emission impact. Duff, originated from detritus or dead planet organic material, is an organic decayed material layer formed on the top of the soil. It is a commonly component of forest or swamp top layer in North American, Europe and Asia.8 In location with warm and moist climate and large forest coverage, deep duff can be formed due to accelerating decomposing of organic material. This is notable in location like southern Appalachians.9 Duff material have different burning emission and flammability features compare to other fuel.7 Duff consumption is only significant in dry burning condition like after wildfire after prolonged drought, due to its limited flammability in moist condition. Simulation study of the 2016 Rough Ridge Fire southern Appalachians estimated duff burning contribute to 60% of the P.M. 2.5 emission from the fire.10 Previous ambient BrC measurements have shown that significant photobleaching effects occurred as it travels away from the source.11 However, aqueous aging of BrC have shown to have a photo-enhancement effect in multiple lab measurements.12–15 It has been shown that the photo-enhancement effect stemmed from the formation of larger chromophores species (oligomer).16 In this work, we investigated direct aqueous photolysis effects on water-soluble biomass burning BrC stemmed from a simulated prescribed and wildfire burning campaign. Representing fuel bed material collected from three forest regions of Georgia (Piedmont, Coastal Plain, Blue Ridge) were burned in both simulated prescribed and wildfire condition to show variance of source and burning condition. Chemical composition of each sample during photolysis process were analyzed with high resolution electrospray ionization mass spectrometry (ESI-HR-MS).

ChatGPT

ChatGPT

Atmospheric aerosols have the capacity to modulate incoming solar radiation through direct scattering and absorption phenomena. Among the diverse aerosol species, Brown Carbon (BrC) is distinguished by its substantial solar radiation absorption, especially within the UV region (280-400 nm). This absorption capability diminishes at longer wavelengths, imparting its characteristic brownish hue. BrC, as a potent light absorbent, is estimated to account for up to 24% of the aerosol-induced warming effect, and can significantly influence tropospheric photochemistry by diminishing the solar UV radiation that reaches the Earth's surface. Upon emission into the atmosphere, the optical and chemical characteristics of BrC may evolve through aging processes in various media including the gas-phase, clouds, or fog. The precise climate impacts of BrC hinge on the aging process it undergoes and its compositional attributes.

A primary source of atmospheric BrC is biomass burning, which occurs in forms such as open vegetation fires or human biofuel burning activities. It's approximated that biomass burning BrC constitutes a substantial 85% of global primary organic aerosol production. The optical traits of Biomass Burning BrC exhibit a pronounced variability depending on the fuel type and conditions of biomass burning. Presently, biomass burning BrC is a source of uncertainty in climate models due to a limited comprehension of its sources, aging processes, and optical attributes. Two focal points of ongoing study are the conditions of prescribed/wildfire and duff burning.

Prescribed burning, a forest management tactic aimed at mitigating the escalating financial and ecological threats posed by wildfires, is chiefly distinguished from wildfire by the moisture content in the fuels; prescribed fires are executed in high moisture content fuel conditions, particularly post-rainfall, unlike wildfires which arise in drier fuel conditions, such as during drought episodes. In the southeastern US, prescribed fires are more prevalent than wildfires, necessitating an assessment of its aerosol emission impacts to forge improved fire management policies aimed at emission reduction.

Duff, originating from detritus or deceased plant organic material, forms an organic decayed material layer atop the soil, commonly constituting the forest or swamp top layers in North America, Europe, and Asia. In locales with warm,

moist climates and extensive forest cover, deep duff layers can form due to accelerated decomposition of organic material, notably in regions like the southern Appalachians. Duff materials exhibit distinct burning emission and flammability characteristics compared to other fuels, with significant consumption occurring only in dry burning conditions, such as post-wildfire following prolonged droughts, owing to its limited flammability in moist conditions.

Prior ambient BrC measurements have revealed substantial photobleaching effects as it disperses from the source. Conversely, lab measurements have demonstrated a photo-enhancement effect during the aqueous aging of BrC, traced back to the formation of larger chromophore species (oligomers).

In this study, we delve into the direct aqueous photolysis effects on water-soluble biomass burning BrC, derived from a simulated prescribed and wildfire burning campaign. Fuel bed material, collected from three distinct forest regions in Georgia (Piedmont, Coastal Plain, Blue Ridge), was incinerated under both simulated prescribed and wildfire conditions to elucidate variances between source and burning conditions. The chemical composition of each sample during the photolysis process was scrutinized using high-resolution electrospray ionization mass spectrometry (ESI-HR-MS).

S

You

you think the rough ridge fire is not nessesary here?

ChatGPT

ChatGPT

Including specific examples like the Rough Ridge Fire can provide readers with concrete instances that illustrate the broader points being made in the paper. However, the necessity of mentioning the Rough Ridge Fire specifically hinges on the context and the goals of your paper.

Here are a couple of considerations that might help you decide:

1. **Relevance to Your Research**: If the Rough Ridge Fire or its study has direct relevance to your research or serves as a foundational or comparative study, it might be beneficial to include it. It could provide context, support your arguments, or help explain the significance of your research.
2. **Focus and Clarity**: Sometimes, including too many specific examples or diverging into subtopics can detract from the main focus of your paper or make the introduction too dense. Ensure that mentioning the Rough Ridge Fire enhances the clarity and focus of your introduction rather than diverting it.
3. **Guidance from Previous Literature or Advisors**: If previous literature frequently references specific fires like the Rough Ridge Fire, or if advisors suggest its inclusion for clarity or context, it might be worth including.
4. **Space and Word Count Constraints**: If you are working within strict space or word count limits, you may need to prioritize information and decide whether mentioning the Rough Ridge Fire is critical to your introduction or if it could be discussed later in the paper or relegated to a footnote.
5. **Contribution to Understanding**: If the mention of the Rough Ridge Fire significantly contributes to the reader's understanding of the issues at hand, it may be worth including.

Ultimately, the decision should align with the overarching goals of your paper and the expectations of your intended readership.

S

You

add a piece saying it is importance to study duff burning aerosol

ChatGPT

ChatGPT

Certainly! Here's a revised segment of your text with the addition regarding the importance of studying duff burning aerosol:

Duff, originating from detritus or deceased plant organic material, forms an organic decayed material layer atop the soil, commonly seen in forest or swamp top layers across North America, Europe, and Asia. In regions with warm, moist climates and extensive forest cover, deep duff layers can form due to accelerated decomposition of organic material, notably in regions like the southern Appalachians. Duff materials exhibit distinct burning emission and flammability characteristics compared to other fuels. Significant consumption occurs only in dry burning conditions, such as post-wildfire following prolonged droughts, due to its limited flammability in moist conditions.

The study of aerosols generated from duff burning is of paramount importance, given their potential to contribute significantly to atmospheric particulate matter, particularly PM2.5, which has known adverse effects on air quality and human health. Furthermore, the distinct chemical composition of duff-derived aerosols may possess unique light-absorbing properties, thereby potentially impacting atmospheric radiative balance and climate. Through comprehensive analysis of duff burning aerosols, a deeper understanding of their chemical, physical, and optical properties can be attained, which is essential for refining climate models and formulating informed environmental policies. This underscores the significance of investigating the aerosol emissions from duff burning, and its implications on atmospheric chemistry and broader climatic interactions.

S

You

Significant consumption occurs only in dry burning conditions, such as post-wildfire following prolonged droughts, due to its limited flammability in moist conditions. by consumption here i mean it is only burnable in those condition

ChatGPT

ChatGPT

Understood. To clarify the meaning of "consumption" in the context of burnability, you might consider revising the sentence as follows:

Significant burning of duff occurs only in dry conditions, such as post-wildfire following prolonged droughts, due to its limited flammability in moist conditions.

S

You

Duff, originating from detritus or deceased plant organic material, forms an organic decayed material layer atop the soil, commonly seen in forest or swamp top layers across North America, Europe, and Asia.8 In locales with warm, moist climates and extensive forest cover, deep duff can be formed due to accelerating decomposing of organic material. This is notable in location like southern Appalachians.9 Duff materials exhibit distinct burning emission and flammability characteristics compared to other fuels.7 Significant burning of duff occurs only in dry conditions, such as wildfire following prolonged droughts, due to its limited flammability in moist conditions.10 Due to the increasing frequency of extreme drought,11 duff burning's climate impact become more and more important.

ChatGPT

ChatGPT

Your revised paragraph succinctly elaborates on the origin and properties of duff, and its susceptibility to burning under dry conditions, particularly during wildfires following prolonged droughts. The added sentence about the increasing frequency of extreme drought effectively emphasizes the growing importance of understanding duff burning's climate impact. This text sets a good foundation for discussing the necessity and implications of studying aerosols generated from duff burning in the broader context of atmospheric chemistry and climate change. If there is any further enhancement or information you would like to include, or if there are additional points you wish to address, feel free to let me know!

S

You

do i need revise on it?

ChatGPT

ChatGPT

The revised paragraph is quite clear and provides a good explanation of duff, its burning conditions, and the importance of studying duff burning due to climate change. However, there's always room for improvement or further clarification depending on the exact focus and style of your research paper. Here are a few suggestions that might help refine the paragraph further:

1. **Language Consistency and Precision**:
   - You might want to ensure consistency in terminology and language use. For instance, consider using either "locales" or "locations" throughout the text for consistency.
   - "Accelerating decomposing of organic material" could be revised to "accelerated decomposition of organic material" for better readability.
2. **Clarification**:
   - If there are particular aspects of duff burning emissions that are of interest (e.g., specific pollutants or aerosol types), mentioning these could provide clearer insight into the significance of the study.
3. **References**:
   - Ensure that all the references are correctly cited and formatted according to the citation style you are following.
4. **Contextual Information**:
   - Depending on the scope of your paper, you might want to provide more contextual information on how duff burning impacts climate or atmospheric composition.
5. **Transition**:
   - Smooth transitions between sentences can help the flow of the paragraph.

Here's a slightly revised version of your paragraph considering the above points:

"Duff, originating from detritus or deceased plant organic material, forms an organic decayed material layer atop the soil, commonly seen in forest or swamp top layers across North America, Europe, and Asia. In locales with warm, moist climates and extensive forest cover, deep duff can form due to accelerated decomposition of organic material, a phenomenon notably observed in locations like the southern Appalachians. Duff materials exhibit distinct burning emission and flammability characteristics compared to other fuels. Significant burning of duff occurs only in dry conditions, such as wildfire following prolonged droughts, owing to its limited flammability in moist conditions. Due to the increasing frequency of extreme drought, the climate impact of duff burning has become progressively more significant, necessitating thorough examination."

These revisions aim to enhance readability, consistency, and clarity. Adjustments should align with the overall style and focus of your research paper, so feel free to modify or disregard these suggestions as per your judgement.

S

You

rewrite this piece : In each location, fuels were collected by raking fine/litter fuels and handpicking woody fuels and placing them into separate bags and later sorted into fuel type categories.

ChatGPT

ChatGPT

In each location, fine/litter fuels were gathered using rakes, while woody fuels were collected by hand. They were then placed into separate bags and subsequently sorted into respective fuel type categories.

S

You

Georgia WIldland fire Simulation Experiment (G-Wise) was conducted in Athens prescribed burn lab (U.S Forest Service Southern Research Station, GA, USA)

between Oct 25 2022 to Nov 19 2022. Fuel burned in this work were collected from Oconee National Forest (Piedmont), southern Blue Ridge mountains and Fort Stuart (Coastal Plain) in Georgia. In each location, fine/litter fuels were gathered using rakes, while woody fuels were collected by hand. They were then placed into separate bags and subsequently sorted into respective fuel type categories. After collection, fuels were condition to preferred moisture level monitoring by a fuel moisture analyzer (Computrack 4000 XL, Brookfield Ametek, MA) though oven drying, submerging in water or placed in humidifier room base on the fuel type. Once desired moisture level achieved, portion of the fuels were weighted and storge in a zip lock bag till the burning day. In the burn day, fuel bed was assembled in a ring representing fuel bed in the wild. The fuel bed break down was summarized in figure 1. Fuels bed was ignited once setup under approximate 2mph wind simulated by a fan. During the burning process, the fire was monitored with a FTIR camera (FLIR A655sc, Teledyne, OR) placed over head of the fuel bed ring. Onced the fuel bed burning have passed smoldering phase confirmed by fuel bed temperature reached below 500oF measured by IR camera, fresh smoke aerosols were collected on 47mm PTFE filter (0.2microns, Sterlitech Corptation). The collected time of each filter varied from 5 to 15min depending the particular concentration in the burn room.

ChatGPT

ChatGPT

The Georgia Wildland Fire Simulation Experiment (G-WISE) was carried out at the Athens prescribed burn lab (U.S. Forest Service Southern Research Station, GA, USA) from October 25 to November 19, 2022. The fuels utilized in this study were sourced from Oconee National Forest (Piedmont), southern Blue Ridge mountains, and Fort Stuart (Coastal Plain) in Georgia. At each location, fine/litter fuels were collected with rakes, while woody fuels were handpicked. These fuels were placed into separate bags and later categorized based on their fuel types. Post-collection, the fuels were conditioned to the desired moisture levels, monitored using a fuel moisture analyzer (Computrack 4000 XL, Brookfield Ametek, MA), through methods such as oven drying, water submersion, or humidifier room placement, contingent on the fuel type. Upon achieving the

desired moisture levels, portions of the fuels were weighed and stored in zip-lock bags until the day of burning.

On the burn day, a fuel bed was assembled within a ring to mimic a natural fuel bed arrangement. The breakdown of the fuel bed is illustrated in Figure 1. Ignition of the fuel bed was carried out under a simulated wind condition of approximately 2 mph, generated by a fan. Throughout the burning process, fire dynamics were monitored using an overhead FTIR camera (FLIR A655sc, Teledyne, OR) placed above the fuel bed ring. Once the burning phase transitioned beyond smoldering, as confirmed by the fuel bed temperature dropping below 500°F as measured by the IR camera, fresh smoke aerosols were collected onto 47mm PTFE filters (0.2 microns, Sterlitech Corporation). The collection duration for each filter ranged between 5 to 15 minutes, depending on the specific concentration levels in the burn room.

S

You

Results found from this work have three main implications: 1) Prescribed and wildfire conditions' impact on biomass burning W-BrC produce are depending on fuel bed. For fuel beds that don't contain duff (Piedmont and Coastal Plain), prescribed burning produced W-BrC with slightly higher AAE as more moisture in fuel results in more smoldering. For fuel beds that contain substantial duff like Blue Ridge, wildfire W-BrC have higher AAE as duff material only contribute to smothering in dryer condition. Though only the water-soluble portion is being analyzed in this work, these results highlight the importance of burning condition to optical properties of primary aerosol produce by biomass burning. When considering climate impact of biomass burning BrC, fuels source and burning condition should be considered together to enable better representation of its impact. 2) Though different biomass burning fuel type and burning condition have different optical properties, among sample investigated in this work, aqueous direct photolysis process render them similarly resulting a unified AAE in the end. This means that potentially biomass burning BrC can be all treated as the same once one or two days in the atmospheric. 3) Both photo-bleaching and photo-enhancement of W-BrC was observed in this work under direct aqueous photolysis. Overall photobleaching is a more important

effect here on the overall absorptivity of change of W-BrC under aqueous phase direct photolysis.

ChatGPT

ChatGPT

The findings from this study elucidate three primary implications:

1. **Influence of Burn Conditions on Water-Soluble Brown Carbon (W-BrC) Production**:
   - The impact of prescribed and wildfire conditions on biomass burning W-BrC production is contingent upon the composition of the fuel bed. In fuel beds devoid of duff (as observed in Piedmont and Coastal Plain), prescribed burning engenders W-BrC

S

You

continued

ChatGPT

ChatGPT

with a slightly elevated Absorption Ångström Exponent (AAE), as the higher moisture content in the fuel facilitates more smoldering. Conversely, in fuel beds with a significant presence of duff, like Blue Ridge, wildfire conditions yield a higher AAE for W-BrC since duff material predominantly contributes to smoldering in drier conditions. While this study analyzes only the water-soluble fraction, the results underscore the crucial role of burn conditions in determining the optical properties of primary aerosols generated by biomass burning. For a more accurate representation of the climatic impact of biomass burning BrC, it is imperative to consider both fuel source and burning conditions in tandem.

2. **Uniformity in AAE Post-Aqueous Direct Photolysis**:
   - Despite varying optical properties stemming from different biomass fuel types and burning conditions, the aqueous direct

photolysis process exhibited a homogenizing effect on the samples investigated in this study, culminating in a unified AAE. This suggests that biomass burning BrC may exhibit similar characteristics after one or two days in the atmosphere, irrespective of their initial diverse properties.

3. **Photo-Bleaching and Photo-Enhancement of W-BrC**:
   - Both photo-bleaching and photo-enhancement phenomena were observed for W-BrC under direct aqueous photolysis in this study. However, photo-bleaching emerged as the more dominant effect, significantly influencing the overall absorptivity changes of W-BrC under aqueous phase direct photolysis.

These implications collectively accentuate the nuanced interplay between fuel composition, burn conditions, and the subsequent atmospheric interactions of biomass burning BrC, enhancing the understanding crucial for refining climate models and environmental policy formulations.

S

You

do it one more time

ChatGPT

ChatGPT

Certainly! Here's another rendition of your findings section:

The outcomes of this study present three primary implications:

1. **Impact of Burning Conditions on Water-Soluble Brown Carbon (W-BrC) Generation**:
   - The effect of both prescribed and wildfire conditions on biomass burning W-BrC production is reliant on the composition of the fuel bed. In scenarios where the fuel bed lacks duff (as seen in Piedmont and Coastal Plain), prescribed burning yields W-BrC with a marginally higher Absorption Ångström Exponent (AAE)

due to increased moisture facilitating more smoldering. On the other hand, in fuel beds with considerable duff content, like Blue Ridge, the W-BrC from wildfire conditions exhibits a higher AAE as duff material predominantly contributes to smoldering in drier conditions. While this analysis focused on the water-soluble segment, the results spotlight the critical role of burning conditions in shaping the optical properties of primary aerosols emanating from biomass burning. To better gauge the climatic repercussions of biomass burning BrC, it's crucial to consider both the fuel source and burning conditions concurrently.

2. **Uniform AAE Post-Aqueous Direct Photolysis**:
    - Despite the initial diversity in optical properties due to different biomass fuel types and burning conditions, the aqueous direct photolysis process in this study demonstrated a unifying effect, resulting in a consolidated AAE across the samples. This insinuates that post one or two days in the atmosphere, biomass burning BrC could potentially exhibit homogenous characteristics regardless of their initial variances.
3. **Photo-Bleaching and Photo-Enhancement Dynamics of W-BrC**:
    - This study observed both photo-bleaching and photo-enhancement of W-BrC under direct aqueous photolysis. However, photo-bleaching appeared to be the more predominant effect, significantly impacting the overall change in absorptivity of W-BrC under aqueous phase direct photolysis.

Collectively, these implications shed light on the intricate interplay between fuel composition, burn conditions, and the subsequent atmospheric interactions of biomass burning BrC, thereby enriching the understanding vital for refining climate models and informing environmental policy frameworks.

S

You

dont use intent

ChatGPT

ChatGPT

Absolutely, here's a revised version of your findings section without indentations:

The discoveries from this research lead to three main implications:

1. **Impact of Burn Conditions on Water-Soluble Brown Carbon (W-BrC) Production**: The effect of prescribed and wildfire conditions on biomass burning W-BrC production varies with the fuel bed composition. For fuel beds without duff (Piedmont and Coastal Plain), prescribed burning produces W-BrC with a slightly higher Absorption Ångström Exponent (AAE) as increased moisture in fuel results in more smoldering. In contrast, for fuel beds with a substantial amount of duff like Blue Ridge, wildfire conditions result in higher AAE for W-BrC as duff material contributes to smoldering mainly in drier conditions. While this work analyzes only the water-soluble portion, the findings highlight the significance of burning conditions on the optical properties of primary aerosols produced by biomass burning. To better comprehend the climate impact of biomass burning BrC, it's essential to consider both fuel source and burning conditions concurrently.
2. **Uniform AAE Following Aqueous Direct Photolysis**: Despite the different optical properties due to varied biomass burning fuel types and burning conditions, the aqueous direct photolysis process demonstrated a harmonizing effect, culminating in a unified AAE across the samples. This suggests that biomass burning BrC may exhibit similar properties after one or two days in the atmosphere, irrespective of their initial differences.
3. **Photo-Bleaching and Photo-Enhancement Phenomena in W-BrC**: Both photo-bleaching and photo-enhancement of W-BrC were observed under direct aqueous photolysis in this study. However, photo-bleaching emerged as the more dominant effect, significantly affecting the overall absorptivity changes of W-BrC under aqueous phase direct photolysis.

Together, these implications delve into the nuanced relationship between fuel composition, burn conditions, and the subsequent atmospheric interactions of biomass burning BrC, enhancing the understanding crucial for refining climate models and environmental policy formulations.

S

You

Biomass burning W-BrC sample in this work start with a similar O/C and H/C, average at 0.4 and 1.32 respectively, and ends at 0.46 and 1.27 after 2 hours photolysis.

ChatGPT

ChatGPT

The Biomass burning Water-Soluble Brown Carbon (W-BrC) samples analyzed in this study initially exhibited similar oxygen to carbon (O/C) and hydrogen to carbon (H/C) ratios, averaging at 0.4 and 1.32 respectively. Following a 2-hour photolysis period, there was a slight alteration in these ratios, with the O/C ratio increasing to 0.46 and the H/C ratio decreasing to 1.27. These changes underscore the dynamic nature of W-BrC's chemical composition under photolytic conditions, potentially influencing its optical properties and interactions within the atmospheric milieu.

S

You

Double bond equivalent (DBE) versus the number of carbon atoms distribution of Blue Ridge wildfire sample, both pre- and post-direct aqueous photolysis, is illustrated in Figure 7. To better visualized the different between, only species that have significant change in abundance (disappear/appear after photolysis or doubled/reduced by half) were plotted. DBE vs C of three reference species conjugated polyene (CxHx+2, DBE = 0.5xC), cata-PAHs (DBE = 0.75xC – 0.5),26 theoretical DBE limit for hydrocarbon (DBE = 0.9xC),27 were also shown. Species possessing a DBE/C slope exceeding the polyene reference (CxHx+2) can potentially contribute to absorption in the visible range as BrC chromophores,

due to their uninterrupted conjugation structure. This method of identifying potential BrC chromophores species using DBE vs C plot was first introduced by Lin et al. (2018) and subsequentially adopt in various HR-MS analysis atmospheric BrC sample.18,26,28,29 As species' DBE/C slope move up in the chart closer to polyene, cata-PAHs, hydrocarbon limit reference respectively, the level of conjugation increased so individual species can potentially have stronger light absorbing properties.30 In figure 7, we can clearly see a decreased in C6-13 species intensity and formation of species near the PAHs reference at C>20 range. In addition, the density of signals inside of the "BrC region" increased. To better indicate this density shift into the BrC region, a "BrC percentage" was calculated by normalizing the sum of signal intensity within the BrC region by the total signal intensity. Result of BrC percentage was shown in Table S2. BrC percentage increase as photolysis progress and majority of the change is happening in the first hour, correlating with the photo-enhancement effect. This DBE vs C analysis approach was previously done in Hopstock et al. (2023) to show condense phase photolysis impacts for primary aerosol collected from urban material burning, a similar trend of depletion of smaller (C6-9) chromophores and increased in larger chromophores (C16-20) signals were found. 18

ChatGPT

ChatGPT

The distribution of Double Bond Equivalent (DBE) versus the number of carbon atoms for the Blue Ridge wildfire sample, both pre- and post-direct aqueous photolysis, is depicted in Figure 7. To enhance visualization of the differences, only species exhibiting significant changes in abundance (either appearing/disappearing post-photolysis or experiencing a doubling/halving in quantity) were plotted. The DBE vs Carbon plot also includes three reference species: conjugated polyene (CxHx+2, DBE = 0.5xC), cata-PAHs (DBE = 0.75xC – 0.5), and the theoretical DBE limit for hydrocarbons (DBE = 0.9xC), providing a comparative framework. Species that exhibit a DBE/C slope surpassing that of the polyene reference (CxHx+2) may potentially contribute to absorption within the visible range as BrC chromophores, owing to their uninterrupted conjugation structure. This innovative approach to identifying potential BrC chromophore species using a DBE vs Carbon plot was pioneered by Lin et al.

(2018) and has been subsequently employed in various High-Resolution Mass Spectrometry (HR-MS) analyses of atmospheric BrC samples. As the DBE/C slope ascends on the plot, nearing the polyene, cata-PAHs, and hydrocarbon limit references, the level of conjugation augments, implying that individual species could potentially exhibit stronger light-absorbing properties. In Figure 7, a discernible decrease in the intensity of C6-13 species and the emergence of species near the PAHs reference at the C>20 range are evident. Moreover, the signal density within the "BrC region" amplified. To better represent this density shift into the BrC region, a “BrC percentage” was computed by normalizing the sum of signal intensity within the BrC region against the total signal intensity. The results of the BrC percentage are tabulated in Table S2, indicating an increase in BrC percentage as photolysis progresses, with the majority of the change transpiring within the first hour, aligning with the photo-enhancement effect observed.

This method of DBE vs Carbon analysis echoes the approach employed by Hopstock et al. (2023) to elucidate the impacts of condensed phase photolysis on primary aerosols derived from urban material burning. A comparable trend, involving the depletion of smaller (C6-9) chromophores and an increase in larger chromophores (C16-20) signals, was discerned, thereby presenting an analogous trajectory in the molecular evolution under photolytic conditions.

S

You

is this paragraphe too long?

ChatGPT

ChatGPT

The paragraph is quite dense and lengthy, which might make it challenging for some readers to digest all the information at once. It's packed with technical details, comparisons, and references to figures and external works. Here are a few strategies you could consider to enhance readability and comprehension:

1. **Break it Down**: You might want to break it down into smaller paragraphs, each focusing on a specific aspect of the analysis or findings.
2. **Use Bullet Points**: For listing multiple points or references, bullet points can make the text easier to read.
3. **Separate Discussion**: If possible, separate the discussion about the findings from the comparison to other studies like Hopstock et al. (2023).
4. **Simplify Language**: Where possible, simplify the language or explain technical terms to make the text more accessible.
5. **Use Visuals**: Ensure that your figures and tables are clear, well-labeled, and directly referenced in the text to help readers understand the data.

Here's how you might break down the original paragraph:

The distribution of Double Bond Equivalent (DBE) versus the number of carbon atoms for the Blue Ridge wildfire sample, both pre- and post-direct aqueous photolysis, is depicted in Figure 7. To enhance visualization of the differences:

- Only species exhibiting significant changes in abundance (either appearing/disappearing post-photolysis or experiencing a doubling/halving in quantity) were plotted.
- The DBE vs Carbon plot also includes three reference species: conjugated polyene (CxHx+2, DBE = 0.5xC), cata-PAHs (DBE = 0.75xC – 0.5), and the theoretical DBE limit for hydrocarbons (DBE = 0.9xC).

Species that exhibit a DBE/C slope surpassing that of the polyene reference (CxHx+2) may potentially contribute to absorption within the visible range as BrC chromophores due to their uninterrupted conjugation structure. ...

This method of DBE vs Carbon analysis echoes the approach employed by Hopstock et al. (2023) to elucidate the impacts of condensed phase photolysis on primary aerosols derived from urban material burning. A comparable trend involving the depletion of smaller (C6-9) chromophores and an increase in larger chromophores (C16-20) signals was discerned, ...

Such division allows for clearer segmentation of ideas and easier digestion of the information presented.

S

You

can we put those history of the method together with hopstock comparson

ChatGPT

ChatGPT

Certainly! Combining the historical context of the method along with the comparison to Hopstock's work could streamline the narrative. Here's a possible way to structure that:

This method of DBE vs Carbon analysis, pioneered by Lin et al. (2018) and subsequently employed in various High-Resolution Mass Spectrometry (HR-MS) analyses of atmospheric BrC samples, provides a robust framework for identifying potential BrC chromophore species. The approach delineates the nuanced molecular evolution under photolytic conditions, spotlighting species with heightened light-absorbing properties based on their DBE/C slopes in relation to reference species.

Echoing this methodological approach, Hopstock et al. (2023) conducted a similar analysis to elucidate the impacts of condensed phase photolysis on primary aerosols derived from urban material burning. A comparable trend was observed, involving the depletion of smaller (C6-9) chromophores and an increase in larger chromophores (C16-20) signals. This parallel underscores the method's utility in dissecting the molecular and optical intricacies of BrC across diverse combustion scenarios and photolytic conditions.

In this manner, the paragraph encapsulates the historical lineage of the method, its application in the current study, and a comparative insight from another significant work in the field, making for a concise and coherent narrative.

S

You

i want to say introducting the method, and probably not the word echoing

ChatGPT

ChatGPT

Absolutely, you can introduce the method and then transition to the comparison with Hopstock et al. (2023) without using the word "echoing." Here's a revised version:

The method of analyzing Double Bond Equivalent (DBE) versus the number of carbon atoms, as introduced by Lin et al. (2018), has been adopted in various High-Resolution Mass Spectrometry (HR-MS) analyses of atmospheric BrC samples. This method provides a significant framework for identifying potential

BrC chromophore species and understanding their molecular evolution under photolytic conditions.

Utilizing this method, Hopstock et al. (2023) explored the impacts of condensed phase photolysis on primary aerosols collected from urban material burning. A similar trend was observed, where smaller chromophores (C6-9) were depleted, and larger chromophores (C16-20) signals increased, demonstrating a consistent molecular behavior across different combustion scenarios and photolytic conditions. This comparison underscores the versatility and relevance of the DBE vs Carbon analysis method in dissecting the molecular and optical intricacies of BrC in diverse environmental settings.

In this version, the method's introduction and its adoption in subsequent studies is clearly stated, followed by a transition to the comparison with Hopstock et al. (2023) to showcase a similar trend observed in different research settings.

S

You

Previously, the photo enhancement effect in UVB aqueous photolysis of furnace generated biomass burning W-BrC have also been investigated with the effect mainly evaluated at 365nm or 400nm.16,22,23 Results from these previous works was summarized in table S2 and our result at these two wavelengths are shown in figure S4. In this work, the photo-enhancement effect at 365nm peaks at 10%-35% increased and 5%-20% increased at 400nm. Observed level was similar compared to previous work at 365 nm (~40%-68%) 16,22 but substantially lower at 400 nm (~150%-235%) 16,22,23. Three factors may contribute to the difference here: 1) Biomass burning W-BrC generated from furnace at set temperature may potentially be different to a realistic setting like open fire conducted in this work. 2) Fuels burned in this work have more diversity compared to 100% firewood used in furnace studies. 3) Wavelength

and intensity of lamps used for photolysis was different compared to previous research. These differences maybe result in different chemistry during aqueous photolysis process. Overall, these different stressed the importance of studying aerosols generated in a more realistic setting.

ChatGPT

ChatGPT

In earlier studies, the photo-enhancement effect during UVB aqueous photolysis of furnace-generated biomass burning W-BrC has been explored, with evaluations primarily conducted at 365nm or 400nm wavelengths. Summaries of these past findings are presented in table S2, while our results for these wavelengths are depicted in figure S4. In the current study, the photo-enhancement effect peaked at an increase of 10%-35% at 365nm, and 5%-20% at 400nm. While the observed level at 365nm aligns relatively well with prior work (~40%-68%), the level at 400nm is markedly lower (~150%-235%). Three contributing factors to these disparities are identified:

1. The biomass burning W-BrC generated in a furnace at set temperatures might inherently differ from those produced in more realistic open fire scenarios, as emulated in this study.
2. The variety of fuels burned in this study is broader compared to the exclusive use of firewood in furnace studies, potentially influencing the resulting W-BrC composition.
3. The wavelength and intensity of lamps employed for photolysis in this study diverged from those used in preceding research, which could lead to variations in the chemistry during the aqueous photolysis process.

Collectively, these distinctions underscore the importance of examining aerosols generated in settings that closely mimic real-world conditions to garner a more accurate understanding of their behavior and implications. This inquiry into more realistic burning conditions, fuel diversity, and photolysis settings not only enriches the comprehension of W-BrC photo-enhancement but also bridges the knowledge gap between controlled laboratory conditions and actual environmental settings.

1 / 2

S

You

Van Krevelen diagram of all samples' abundance weighted average composition progression during aqueous photolysis experiment. Overall, composition of sample starts at H/C: 0.4 and O/C:1.32 and ends at H/C: 0.46 and O/C:1.27 after 2 hours aqueous photolysis.

ChatGPT

ChatGPT

The Van Krevelen diagram, utilized to trace the abundance-weighted average composition progression of all samples during the aqueous photolysis experiment, reveals a discernible shift in the H/C and O/C ratios over the 2-hour photolysis period. Initially, the composition of the samples is characterized by H/C and O/C ratios of 0.4 and 1.32 respectively. Post the 2-hour aqueous photolysis, these ratios undergo a modification, with the H/C ratio increasing to 0.46, while the O/C ratio decreases to 1.27. This graphical depiction encapsulates the molecular evolution experienced by the samples under photolytic conditions, offering insight into the chemical dynamics that govern the behavior of Water-Soluble Brown Carbon (W-BrC) in aqueous environments. The shifts in H/C and O/C ratios, as illustrated by the Van Krevelen diagram, underscore the intricacies of the chemical transformations, potentially influencing the optical and atmospheric properties of the W-BrC.

S

You

The effect of prescribed and wildfire conditions on biomass burning W-BrC production varies with the fuel bed composition. For fuel beds without duff (Piedmont and Coastal Plain), prescribed burning produces W-BrC with a slightly higher Absorption Ångström Exponent (AAE) as increased moisture in fuel results in more smoldering. In contrast, for fuel beds with a substantial amount of duff like Blue Ridge, wildfire conditions result in higher AAE for W-BrC as duff material contributes to smoldering mainly in drier conditions. While

this work analyzes only the water-soluble portion, the findings highlight the significance of burning conditions on the optical properties of primary aerosols produced by biomass burning. To better comprehend the climate impact of primary biomass burning BrC, it's essential to consider both fuel source and burning conditions concurrently. summarized this part for a one line in powerpoint

ChatGPT

ChatGPT

Burning conditions affect W-BrC production's Absorption Ångström Exponent (AAE), with prescribed burning in duff-free fuel beds yielding slightly higher AAE due to more smoldering, while wildfire in duff-rich beds like Blue Ridge shows higher AAE; understanding both fuel type and burning conditions is crucial for assessing climate impacts of primary biomass burning BrC.

S

You

Both monomer were reported to be capable of undergo oligomerization in aqueous phase through photo-oxidation or acid-catalyzed. i have two monomer here, so each one only go through one of these mechanism.

ChatGPT

ChatGPT

Each monomer is reported to undergo oligomerization in the aqueous phase, one through photo-oxidation and the other through acid-catalysis.

S

You

photo-oxi is for vannlic acid and the other is levoglucosan

ChatGPT

ChatGPT

Vanillic acid undergoes oligomerization via photo-oxidation, while levoglucosan proceeds through acid-catalysis in the aqueous phase.

S

You

add is reported to

ChatGPT

ChatGPT

Vanillic acid is reported to undergo oligomerization via photo-oxidation, while levoglucosan is reported to proceed through acid-catalysis in the aqueous phase.

S

You

Figures 6 and S5-10 display the assigned signals and mass spectra of all samples collected in this study. A comparison of the mass spectra of Blue Ridge wildfire W-BrC before and after photolysis reveals a notable formation of a regular pattern in the > 215m/z region after photolysis. Within this region, clusters of signals are separated by 12, 14, 16 or 18 u while prominent peaks within each cluster separated by 2 u. The formation of the regular mass spectra peaks pattern with repeating unit is an indication of oligomer formations. In addition, two monomer vanillic acid (C8H8O4) and levoglucosan (C6H10O5) and their corresponding oligomer were highlighted in figure 6. Notable suppression of the highlight monomer signal and enhancement of oligomer signal after photolysis. Vanillic acid was reported can undergo oligomerization via photo-oxidation,25 while levoglucosan is reported to proceed through acid-catalysis in the aqueous phase.26 Both the formation of regular mass spectra pattern and these individual signals changed are clear evidence of oligomer formation during aqueous photolysis process.

ChatGPT

ChatGPT

Figures 6 and S5-10 exhibit the assigned signals and mass spectra of all samples collected in this study. A scrutiny of the mass spectra for Blue Ridge wildfire W-BrC, both pre and post-photolysis, unveils a pronounced formation of a regular pattern in the >215m/z region following photolysis. Within this domain, clusters of signals are demarcated by 12, 14, 16, or 18 u, while notable peaks within each cluster are separated by 2 u. This emergence of a regular mass spectra peaks pattern with repeating units signals the formation of oligomers. Furthermore, two monomers, vanillic acid ($C8H8O4$) and levoglucosan ($C6H10O5$), along with their respective oligomers, are spotlighted in Figure 6. A significant suppression of the highlighted monomer signals and an enhancement of the oligomer signals post-photolysis are observed. Vanillic acid is reported to undergo oligomerization via photo-oxidation, while levoglucosan is reported to proceed through acid-catalysis in the aqueous phase. Both the establishment of a regular mass spectra pattern and the alterations in these individual signals serve as clear evidence of oligomer formation during the aqueous photolysis process.

S

You

So i also have a figure that instead of shown the high mass spect, i just shown the C8, C16 C24 species so reader can see the intensified of oligomer species, how to introduce this chart.

ChatGPT

ChatGPT

In addition to the detailed mass spectra, Figure X provides a focused view on the intensification of oligomer species by showcasing only the C8, C16, and C24 species across the samples. This narrowed perspective accentuates the progressive oligomerization process by visually representing the amplification of oligomer species, thus offering a clear illustration of the oligomerization trajectory. Through this simplified representation, readers can easily discern the burgeoning of oligomer species, underscoring the substantial role of aqueous photolysis in driving oligomer formation from the monomeric counterparts.

S

You

Do this as real text in the paper

ChatGPT

ChatGPT

Certainly! Here's a revised text that could fit into your paper:

To provide a clearer illustration of the oligomerization process, Figure X presents a simplified view by exclusively showcasing the C8, C16, and C24 species across the samples. This focused representation accentuates the intensification of oligomer species, offering a visually succinct depiction of the oligomerization trajectory. By honing in on these specific species, the progressive amplification of oligomer species through the stages of aqueous photolysis is vividly demonstrated, underscoring the pivotal role of the photolytic conditions in driving the transition from monomeric to oligomeric forms. Through this streamlined depiction, the substantiation of oligomer formation, a central theme of this investigation, is rendered more accessible and discernible to the readers, thereby enhancing the elucidation of the underlying processes governing the oligomerization under varying photolytic conditions.

S

You

also i calculated the integrated 215-600 over 100-215 signal to shown the increased of portion of signal from oligomer region. how to introduce this also.

ChatGPT

ChatGPT

To further quantify the progression of oligomerization, the ratio of integrated signals from the oligomer region (215-600 m/z) to that of the lower mass region

(100-215 m/z) was calculated. This ratio, depicted in Figure Y, provides a quantitative measure of the relative increase in the oligomer signal intensity, offering a more nuanced insight into the oligomerization dynamics. By examining this ratio, the augmentation of the oligomer species' signal, indicative of the extent of oligomerization, can be systematically evaluated. This metric, paired with the visual illustrations provided earlier, furnishes a comprehensive understanding of the oligomerization process across the different stages of aqueous photolysis, elucidating the shift in signal intensity from the lower mass region to the oligomer region. Through this analytical lens, the interplay between the photolytic conditions and the ensuing oligomer formation is further illuminated, providing a robust framework for dissecting the molecular transformations underpinning the observed oligomerization phenomena.

S

You

this is too long, i have a table not a figure.

ChatGPT

ChatGPT

To quantitatively illustrate the increase in oligomer signal intensity, a ratio was calculated by integrating the signal from the oligomer region (215-600 m/z) over that of the lower mass region (100-215 m/z). This ratio, tabulated in Table Z, succinctly quantifies the heightened presence of oligomer species, shedding light on the extent of oligomerization as influenced by the photolytic conditions.

S

You

connect these three piece together

ChatGPT

ChatGPT

Certainly! Here's a revised and connected version of your text based on the three pieces provided:

To elucidate the oligomerization process further, Figure X hones in on the intensification of oligomer species by exclusively showcasing the C8, C16, and C24 species across the samples. This focused view accentuates the oligomerization trajectory, offering a visually succinct depiction of the progressive amplification of oligomer species through the stages of aqueous photolysis.

Furthermore, a quantitative lens is also employed to evaluate the oligomerization dynamics. A ratio was computed by integrating the signal from the oligomer region (215-600 m/z) over that of the lower mass region (100-215 m/z). This ratio, tabulated in Table Z, succinctly quantifies the heightened presence of oligomer species, shedding light on the extent of oligomerization as influenced by the photolytic conditions.

Collectively, the visual representation in Figure X, paired with the quantitative analysis provided in Table Z, furnishes a comprehensive understanding of the oligomerization process. This dual approach—visual and quantitative—enables a robust evaluation of the molecular transformations driving oligomer formation under varying photolytic conditions, thereby enhancing the elucidation of the oligomerization phenomena central to this investigation.

S

You

no i mean just the figure 6 piece. and these two. also dont make conclusion

ChatGPT

ChatGPT

Certainly! Here's a connected version of the three pieces concerning Figure 6, the oligomer species chart, and the integrated signal ratio in Table Z:

Figures 6 and S5-10 display the assigned signals and mass spectra of all samples collected in this study. A comparison of the mass spectra of Blue Ridge wildfire W-BrC before and after photolysis reveals a notable formation of a regular pattern in the > 215m/z region after photolysis. Within this region, clusters of signals are separated by 12, 14, 16 or 18 u while prominent peaks within each cluster separated by 2 u. The formation of the regular mass spectra peaks pattern with repeating units is an indication of oligomer formations. In addition, two monomers vanillic acid ($C_8H_8O_4$) and levoglucosan ($C_6H_{10}O_5$) and their corresponding oligomer were highlighted in figure 6. Notable suppression of the highlighted monomer signal and enhancement of oligomer signal after photolysis are observed. Vanillic acid is reported to undergo oligomerization via photo-oxidation, while levoglucosan is reported to proceed through acid-catalysis in the aqueous phase.

To provide a clearer illustration of the oligomerization process, a separate chart focuses exclusively on showcasing the C8, C16, and C24 species across the samples. This focused representation accentuates the intensification of oligomer species, offering a visually succinct depiction of the oligomerization trajectory.

Furthermore, to quantitatively illustrate the increase in oligomer signal intensity, a ratio was calculated by integrating the signal from the oligomer region (215-600 m/z) over that of the lower mass region (100-215 m/z). This ratio, tabulated in Table Z, succinctly quantifies the heightened presence of oligomer species, shedding light on the extent of oligomerization as influenced by the photolytic conditions.

S

You

To investigate chemical evolution through direct aqueous photolysis, offline ESI-MS analysis was performed. Elementals group distribution of each unaged samples was summarized in figure 5. Formula detected and assigned for all sample are almost all CHO and CHNO with sparse CH and CHN. Significantly more species were detected in Blue Ridge sample (4634/3090 for wildfire/prescribed) compared to all other sample which have around 3000 species. Also, Blue Ridge sample have higher percent of N containing species. This result shown higher level of chemical complexity due to duff burning compared to other fuel. Abundance weighted average of C/O, C/H ratio of each sample were calculated and shown in figure 5 to show the overall composition change of photolysis. The samples analyzed in this study initially exhibited similar oxygen to carbon (O/C) and hydrogen to carbon (H/C) ratios, averaging at 0.4 and 1.32 respectively. Following a 2-hour photolysis period, there was a slight alteration in these ratios, with the O/C ratio increasing to 0.46 and the H/C ratio decreasing to 1.27. Overall, aqueous photolysis in this work result in an increased C/O indicating oxidation decreased in C/H indicating oxidation. 24 However, in our case, these changed observed are minimal and don't result in a overall big shift in chemical composition of W-BrC studies. Figures 6 and S5-10 display the assigned signals and mass spectra of all samples collected in this study. A comparison of the mass spectra of Blue Ridge wildfire W-BrC before (Figure 6(a), (b)) and after photolysis (Figure 6(c), (d)) reveals a notable suppression of the C8H8O4 signal (vanillic acid) and an enhancement of the C16H14O8 (possible vanillic acid dimer), C24H22O16 (possible vanillic acid trimer) peak post-photolysis. Phenolic monoaromatic species, such as vanillic acid or guaiacol, can undergo photooxidation reactions, forming oligomers.25 These oligomers are also BrC and have observed to be less reactive and have lifetime in aqueous photolysis compared to their smaller counterpart.22 List of possible monoaromatic species detected in each spectra was summarized in Table S3. To elucidate the oligomer formation, we segmented the mass spectra into a monomer range (100-215 m/z) and an oligomer range (215-600 m/z), integrating the mass spectral signal intensity in both ranges to compute the oligomer-to-monomer ratio. This ratio serves as an indicator of oligomer production and monomer depletion. A summary of the ratios from all tested samples is provided in Table 1. During photolysis, this ratio increased

significantly, with most of the increase occurring within the first hour, correlating with the photo-enhancement effect. Double bond equivalent (DBE) versus the number of carbon atoms distribution of Blue Ridge wildfire sample, both pre- and post-direct aqueous photolysis, is illustrated in Figure 7. To better visualized the different between, only species that have significant change in abundance (disappear/appear after photolysis or doubled/reduced by half in abundance) were plotted. DBE vs C of three reference species conjugated polyene (CxHx+2, DBE = 0.5xC), cata-PAHs (DBE = 0.75xC – 0.5),26 theoretical DBE limit for hydrocarbon (DBE = 0.9xC),27 were also shown. Species possessing a DBE/C slope exceeding the polyene reference (CxHx+2) can potentially contribute to absorption in the visible range as BrC chromophores, due to their uninterrupted conjugation structure. 28 As species' DBE/C slope move up in the chart closer to polyene, cata-PAHs, hydrocarbon limit reference respectively, the level of conjugation increased so individual species can potentially have stronger light absorbing properties. In figure 7, we can clearly see a decreased in C6-13 species intensity and formation of species near the PAHs reference at C>20 range. In addition, the density of signals inside of the "BrC region" increased. To better indicate this density shift into the BrC region, a "BrC percentage" was calculated by normalizing the sum of signal intensity within the BrC region by the total signal intensity. Result of BrC percentage was shown in Table 1. BrC percentage increase as photolysis progress and majority of the change is happening in the first hour, correlating with the photo-enhancement effect. This result combined with photo-enhancement effect observed in visible, suggested that the aqueous photolysis process is potentially producing larger BrC chromophores. The approach of identifying potential BrC chromophores with Double Bond Equivalent (DBE) versus the number of carbon atoms plot, as introduced by Lin et al. (2018), has been adopted in various HR-MS analyses of atmospheric BrC samples. 26,29–31 Utilizing this method, Hopstock et al. (2023) explored the impacts of condensed phase photolysis on primary aerosols collected from urban material burning. A similar trend was observed, where smaller chromophores (C6-9) were depleted, and larger chromophores (C16-20) signals increased.29 A caveat to note is that peak abundance in FT-ICR MS does not linearly relate to the concentration of corresponding species due to issues like differing ionization efficiencies among species and matrix effects.32 Therefore, the intensity-weighted metric derived from FT-ICR MS serves merely as a demonstration for the trend, not as a rigid quantification. In addition to

non-linearity of FT-ICR response, our approache is also bias toward species that have high ionization efficiency under ESI-(-) mode, so species like PAHs that can’t be ionize efficiently in ESI-(-) weren’t account in this assessment.

ChatGPT

ChatGPT

The narrative you've provided delineates a comprehensive investigation of chemical evolution through direct aqueous photolysis, emphasizing the exploration of oligomerization processes. It demonstrates a structured attempt to utilize both visual and quantitative methods to articulate the chemical transformations occurring. Here are a few suggestions to further refine the text, ensuring clarity and coherence:

1. **Clarity and Precision**:
   - Ensure that the terminology and abbreviations are well-defined at their first occurrence to maintain clarity for readers unfamiliar with the subject matter.
   - It might be useful to provide a brief explanation or reference for technical terms such as the Double Bond Equivalent (DBE), to enhance understanding.
2. **Consistency**:
   - Maintain consistency in the representation of formulas, units, and references throughout the text.
   - Ensure consistent capitalization and formatting, especially in figures, tables, and references.
3. **Visual Representations**:
   - Make sure that all figures and tables are well-labeled, and the captions provide a clear description of what is being presented.
4. **Coherence**:
   - Ensure a logical flow of information by organizing the findings in a manner that builds upon the previously stated information.
5. **Analytical Reflection**:
   - A brief discussion on the implications of the findings and how they contribute to the existing body of knowledge might provide a more holistic understanding of the study's impact.

6. **Technical Details**:
   - Consider explaining the significance of the specific m/z ranges chosen for analysis and how they relate to the overarching investigation of oligomerization processes.
7. **Limitations**:
   - The acknowledgment of the limitations associated with the FT-ICR MS technique and the biases toward species with high ionization efficiency in ESI-(-) mode is crucial. You may want to elaborate a bit more on how these limitations could potentially affect the interpretation of the results and the conclusions drawn from this study.
8. **Grammar and Syntax**:
   - Proofread the text for grammatical accuracy and ensure correct syntax to

S

You

To investigate chemical evolution through direct aqueous photolysis, offline ESI-MS analysis was performed. Elementals group distribution of each unaged samples was summarized in figure 5. Formula detected and assigned for all sample are almost all CHO and CHNO with sparse CH and CHN. Significantly more species were detected in Blue Ridge sample (4634/3090 for wildfire/prescribed) compared to all other sample which have around 3000 species. Also, Blue Ridge sample have higher percent of N containing species. This result shown higher level of chemical complexity due to duff burning compared to other fuel. Abundance weighted average of C/O, C/H ratio of each sample were calculated and shown in figure 5 to show the overall composition change of photolysis. The samples analyzed in this study initially exhibited similar oxygen to carbon (O/C) and hydrogen to carbon (H/C) ratios, averaging at 0.4 and 1.32 respectively. Following a 2-hour photolysis period, there was a slight alteration in these ratios, with the O/C ratio increasing to 0.46 and the H/C ratio decreasing to 1.27. Overall, aqueous photolysis in this work result in an increased C/O indicating oxidation decreased in C/H indicating oxidation. 24 However, in our case, these changed observed are minimal and don't result in a overall big shift in chemical composition of W-BrC studies. Figures 6 and S5-

10 display the assigned signals and mass spectra of all samples collected in this study. A scrutiny of the mass spectra for Blue Ridge wildfire W-BrC, both pre and post-photolysis, unveils a pronounced formation of a regular pattern in the >215m/z region following photolysis. Within this domain, clusters of signals are demarcated by 12, 14, 16, or 18 u, while notable peaks within each cluster are separated by 2 u. This emergence of a regular mass spectra peaks pattern with repeating units signals the formation of oligomers. Furthermore, two monomers, vanillic acid ($C_8H_8O_4$) and levoglucosan ($C_6H_{10}O_5$), along with their respective oligomers, are spotlighted in Figure 6. A significant suppression of the highlighted monomer signals and an enhancement of the oligomer signals post-photolysis are observed. Vanillic acid was reported can undergo oligomerization via photo-oxidation,25 while levoglucosan is reported to proceed through acid-catalysis in the aqueous phase.26 To provide a clearer illustration of the oligomerization process, figure 7 focuses exclusively on showcasing the C8, C16, and C24 species across the samples. This focused representation accentuates the intensification of C16, C24 oligomer species, offering a visually succinct depiction of the oligomerization trajectory. Furthermore, to quantitatively illustrate the increase in oligomer signal intensity, oligomer-to-monomer ratio was calculated by integrating the signal from the oligomer region (215-600 m/z) over that of the lower mass region (100-215 m/z). This ratio serves as an indicator of oligomer production and monomer depletion. A summary of the ratios from all tested samples is provided in Table 1. During photolysis, this ratio increased significantly, with most of the increase occurring within the first hour, correlating with the peaking of the photo-enhancement effect. Double bond equivalent (DBE) versus the number of carbon atoms distribution of Blue Ridge wildfire sample, both pre- and post-direct aqueous photolysis, is illustrated in Figure 8. To better visualized the different between, only species that have significant change in abundance (disappear/appear after photolysis or doubled/reduced by half) were plotted. DBE vs C of three reference species conjugated polyene (C_xH_{x+2}, DBE = 0.5xC), cata-PAHs (DBE = 0.75xC – 0.5),27 theoretical DBE limit for hydrocarbon (DBE = 0.9xC),28 were also shown. Species possessing a DBE/C slope exceeding the polyene reference (C_xH_{x+2}) can potentially contribute to absorption in the visible range as BrC chromophores, due to their uninterrupted conjugation structure. As species' DBE/C slope move up in the chart closer to polyene, cata-PAHs, hydrocarbon limit reference respectively, the level of conjugation

increased so individual species can potentially have stronger light absorbing properties.29 In figure 7, we can clearly see a decreased in C6-13 species intensity and formation of species near the PAHs reference at C>20 range. In addition, the density of signals inside of the “BrC region” increased. To better indicate this density shift into the BrC region, a “BrC percentage” was calculated by normalizing the sum of signal intensity within the BrC region by the total signal intensity. Result of BrC percentage was shown in Table 1. BrC percentage increase as photolysis progress and majority of the change is happening in the first hour similar to the oligomer-to-monomer ratio. This result combined with photo-enhancement effect observed in visible, suggested that the aqueous photolysis process is potentially producing larger BrC chromophores. The approach of identifying potential BrC chromophores with Double Bond Equivalent (DBE) versus the number of carbon atoms plot, as introduced by Lin et al. (2018), has been adopted in various HR-MS analyses of atmospheric BrC samples. 27,30–32 Utilizing this method, Hopstock et al. (2023) explored the impacts of condensed phase photolysis on primary aerosols collected from urban material burning. A similar trend was observed, where smaller chromophores (C6-9) were depleted, and larger chromophores (C16-20) signals increased.30 A caveat to note is that peak abundance in FT-ICR MS does not linearly relate to the concentration of corresponding species due to issues like differing ionization efficiencies among species and matrix effects.33 Therefore, the intensity-weighted metric derived from FT-ICR MS serves merely as a demonstration for the trend, not as a rigid quantification. In addition to non-linearity of FT-ICR response, our approache is also bias toward species that have high ionization efficiency under ESI-(-) mode, so species like PAHs that can’t be ionize efficiently in ESI-(-) weren’t account in this assessment.

ChatGPT

ChatGPT

Your narrative provides a detailed exposition of the research conducted. It seems to be meticulously organized and structured, demonstrating a solid attempt to articulate complex analytical processes and findings. Here are a few suggestions that might help in further refining your manuscript:

1. **Terminology and Abbreviations**:

- Ensure that all terms and abbreviations are well-defined upon their first occurrence to maintain clarity for readers who might not be familiar with the subject matter.

2. **Grammar and Syntax**:
   - Proofreading for grammatical accuracy and correct syntax is essential. For instance, phrases like "Elementals group distribution of each unaged samples was summarized..." could be revised for grammatical accuracy.
3. **Numerical Values and Units**:
   - Ensure consistency in representing numerical values and units. It's good practice to follow standard conventions in the representation of units and values.
4. **Visual Representations**:
   - Ensure all figures and tables are well-labeled, and the captions provide a clear description of what is being presented. It's also essential to ensure that references to figures and tables in the text match the actual figure and table numbers.
5. **Methodological Clarity**:
   - It could be beneficial to explain the significance of certain methodological choices or parameters, especially if they are central to the findings of your study.
6. **Logical Flow**:
   - Ensure a logical flow of information by organizing the findings in a manner that builds upon the previously stated information. Each paragraph should transition smoothly to the next, maintaining a coherent narrative.
7. **Discussion of Limitations**:
   - The acknowledgment of the limitations associated with the FT-ICR MS technique and the biases toward species with high ionization efficiency in ESI-(-) mode is crucial. It might be beneficial to elaborate a bit more on how these limitations could potentially affect the interpretation of the results and the conclusions drawn from this study.
8. **Technical Details**:

- Provide sufficient context or references for technical details to ensure clarity and comprehension. For example, explaining the significance of the specific m/z ranges chosen for analysis and how they relate to the overarching investigation of oligomerization processes could be helpful.

9. **Conclusions and Implications**:
   - A brief discussion on the implications of the findings and how they contribute to the existing body of knowledge might provide a more holistic understanding of the study's impact.

These suggestions aim to enhance the clarity, coherence, and overall quality of your manuscript, making it more accessible and informative to your readers.

S

You

Bulk chemical composition analysis was carried out on before photolysis experiment, middle of photolysis experiment, end of 2 hours photolysis experiment sample for each fuel type and burning condition. The middle point of photolysis was chosen to be the peak or near the peak of the photo-enhancement effect based on UV-Vis observation. ESI-MS analysis was performed on a Bruker SolariX XR 12 T Fourier-Transform Ion Cyclotron Resonance (FT-ICR) mass spectrometer in negative mode over 100-600 m/z range. Peaks assignment of resulting mass spectra was done using the open-source R package MFassignR (Schum et al.2020).18 For each mass spectra, sample noise was removed using default KMDNoise function estimation with a signal to noise cut off of S/N>= 3. Assigned peak lists were extracted following MFAssignR’s isotope filtering and internal mass calibration steps. Assignments in this work were all done with element constraint of O<= 40, N<= 3, S<=1 and limit to singly charge species. Also, background subtraction was done by removing peaks assigned in a water blank processed in the same way. After formula assignment, O/C, H/C, double bond equivalent (DBE),19 was calculated for all formula being identified in all mass spectra collected. DBE for CcHhOoNnSs molecule is calculated as: DBE=c-0.5*h+0.5*n+1 (1) DBE is equal to the sum of C=C, C=O bonds and rings in a molecule.

ChatGPT

ChatGPT

The section you've provided outlines the methodology followed for the bulk chemical composition analysis at different stages of the photolysis experiment, utilizing ESI-MS analysis, and the subsequent data processing and formula assignment processes. Here are a few recommendations that might help refine this section further:

1. **Clarity and Precision**:
    - It's good to see that you've provided a clear sequence of steps followed in the analysis. Maintaining this level of clarity and precision throughout the manuscript will be beneficial for the reader.
2. **Detailing**:
    - You might want to ensure that all technical terms and abbreviations are adequately defined, and references are correctly cited.
3. **Instrumentation**:
    - Detailing the specific settings and configurations of the Bruker SolariX XR 12 T Fourier-Transform Ion Cyclotron Resonance (FT-ICR) mass spectrometer used might be useful. This can include any relevant parameters or conditions that were controlled during the analysis.
4. **Software Tools**:
    - It's great that you've mentioned the software tool (MFassignR) used for peak assignment. It might also be beneficial to provide a brief description or reference for KMDNoise function estimation if it is a significant part of the data processing.
5. **Formula Constraints**:
    - Mentioning the element constraints is good practice. It might also be useful to explain why these particular constraints were chosen if it significantly impacts the analysis.
6. **Equation Formatting**:

- Ensure the equation for DBE is formatted correctly and consistently throughout the manuscript. It might also be beneficial to explain the terms within the equation if they have not been defined earlier.

7. **Interpretation**:
   - Providing brief interpretations or explanations for why specific steps were taken (e.g., why the middle point of photolysis was chosen, or why a particular S/N cutoff was used) might provide the reader with a better understanding of the methodology.
8. **Visual Aids**:
   - If possible, consider including figures or tables that can help visualize the methodology or the results obtained from this analysis. Visual aids can provide a clearer understanding of the technical processes involved.
9. **Cross-referencing**:
   - Ensure that all figures, tables, and equations are correctly cross-referenced within the text, facilitating easier navigation for the reader.
10. **Proofreading**:
    - A thorough proofreading for grammatical accuracy and consistency in terminology and formatting will enhance the overall readability and professionalism of the manuscript.

These suggestions aim to enhance the comprehensibility and coherence of the methodology section, making it easier for readers to follow the procedures and understand the rationale behind specific methodological choices.

S

You

do burning firewood in wet condition result in more smoldering

ChatGPT

ChatGPT

Yes, burning firewood in wet or damp conditions can result in more smoldering as compared to burning it in dry conditions. Here are the main reasons why:

1. **Reduced Combustion Efficiency**:
   - Wet or damp firewood burns less efficiently than dry wood. The moisture in the wood needs to evaporate before the wood can burn, which consumes heat and slows down the combustion process.
2. **Lower Temperatures**:
   - The presence of moisture lowers the temperature of the fire. A lower temperature can lead to incomplete combustion, which is characteristic of smoldering.
3. **Smoldering Phase**:
   - Smoldering is a type of combustion that occurs in the absence of flame and at lower temperatures. The conditions created by burning wet or damp firewood are conducive for smoldering, as there's a reduced availability of heat to sustain flaming combustion.
4. **Reduced Airflow**:
   - Wet or damp conditions can also hinder airflow to the fire, which is essential for sustaining flaming combustion. Reduced airflow can lead to a more prolonged smoldering phase.
5. **Increased Smoke Production**:
   - Smoldering combustion tends to produce more smoke and particulate matter. When burning wet or damp firewood, you may notice an increase in smoke, which is a characteristic of smoldering combustion.
6. **Char Formation**:
   - Smoldering combustion often results in char formation rather than ash. This is another characteristic that is likely to be observed when burning wet or damp firewood.

For optimal burning and reduced smoldering, it's advisable to use dry, seasoned firewood. This will ensure a hotter, more efficient fire with less smoke and fewer particulate emissions.

S

You

This work explores the direct aqueous photolysis process of water-soluble brown carbon (W-BrC) arising from biomass burning, utilizing fuel collected from three distinct regions in Georgia as a part of the Georgia Wildland fire Simulation Experiment(G-Wise) campaign: Piedmont, Coastal Plain, and Blue Ridge, under simulated prescribed and wildfire burning conditions.

ChatGPT

ChatGPT

Your introduction captures the essence of the research quite effectively. However, to enhance clarity and context, consider the following revised version:

"This study delves into the direct aqueous photolysis of water-soluble brown carbon (W-BrC) produced from biomass burning. The research is a segment of the Georgia Wildland Fire Simulation Experiment (G-Wise), focusing on fuel samples collected from three diverse geographical regions in Georgia: the Piedmont, Coastal Plain, and Blue Ridge. These samples were subjected to simulated burning conditions that mimicked both prescribed burns and wildfires. This approach aims to shed light on the photolytic behavior of W-BrC in various environmental conditions and from different fuel sources, contributing to a deeper understanding of its impact on atmospheric chemistry and climate dynamics."

This revision provides a more comprehensive overview of the study's scope, objectives, and the significance of the research in understanding the environmental implications of W-BrC.

S

You

This is parts of a abstract so i want to keep it short

ChatGPT

ChatGPT

Understood, for a concise abstract introduction, you might consider:

"This study investigates the direct aqueous photolysis of water-soluble brown carbon (W-BrC) from biomass burning, analyzing fuels from Georgia's Piedmont, Coastal Plain, and Blue Ridge regions under simulated prescribed and wildfire conditions. The research, part of the Georgia Wildland Fire Simulation Experiment (G-Wise), aims to enhance understanding of W-BrC's atmospheric impacts."

This version succinctly captures the essence of your study while providing a brief context for your research.

S

You

i dont need to aim to part, but still say this is part of G-wise

ChatGPT

ChatGPT

Understood, here's a revised version:

"This study delves into the direct aqueous photolysis of water-soluble brown carbon (W-BrC) derived from biomass burning. It focuses on fuels from Georgia's Piedmont, Coastal Plain, and Blue Ridge areas, subjected to simulated prescribed and wildfire conditions, as part of the Georgia Wildland Fire Simulation Experiment (G-Wise)."

S

You

This work explores the direct aqueous photolysis process of water-soluble brown carbon (W-BrC) arising from biomass burning, utilizing fuel collected from three distinct regions in Georgia: Piedmont, Coastal Plain, and Blue Ridge,

under simulated prescribed and wildfire burning conditions. this is what i have before. i want to keep the same tone just adding the G-wise

ChatGPT

ChatGPT

Certainly! Here's your revised sentence with the addition of G-Wise:

"This work, as part of the Georgia Wildland Fire Simulation Experiment (G-Wise), explores the direct aqueous photolysis process of water-soluble brown carbon (W-BrC) arising from biomass burning. It utilizes fuel collected from three distinct regions in Georgia: Piedmont, Coastal Plain, and Blue Ridge, under simulated prescribed and wildfire burning conditions."

ChatGPT can make mistakes. Consider checking important information.

References:

(1) Madronich, S. *ACOM: Quick TUV*. National Center for Atmospheric Research Atmospheric Chemistry Observations and Modeling. https://www.acom.ucar.edu/Models/TUV/Interactive_TUV/ (accessed 2024-01-02).

(2) Xia, J.-B.; Zhu, C.; Chen, C. Visible Light-Promoted Metal-Free C–H Activation: Diarylketone-Catalyzed Selective Benzylic Mono- and Difluorination. *J Am Chem Soc* **2013**, *135* (46), 17494–17500. https://doi.org/10.1021/ja410815u.

(3) Lignell, H.; Epstein, S. A.; Marvin, M. R.; Shemesh, D.; Gerber, B.; Nizkorodov, S. Experimental and Theoretical Study of Aqueous Cis-Pinonic Acid Photolysis. *J Phys Chem A* **2013**, *117* (48), 12930–12945. https://doi.org/10.1021/jp4093018.

(4) Bunce, N. J.; Lamarre, J.; Vaish, S. P. PHOTOREARRANGEMENT OF AZOXYBENZENE TO 2-HYDROXYAZOBENZENE: A CONVENIENT CHEMICAL ACTINOMETER*. *Photochem Photobiol* **1984**, *39* (4), 531–533. https://doi.org/10.1111/j.1751-1097.1984.tb03888.x.

(5) Wong, J. P. S.; Tsagkaraki, M.; Tsiodra, I.; Mihalopoulos, N.; Violaki, K.; Kanakidou, M.; Sciare, J.; Nenes, A.; Weber, R. J. Atmospheric Evolution of Molecular-Weight-Separated Brown Carbon from Biomass Burning. *Atmos Chem Phys* **2019**, *19* (11), 7319–7334. https://doi.org/10.5194/acp-19-7319-2019.

(6) Hems, R. F.; Schnitzler, E. G.; Bastawrous, M.; Soong, R.; Simpson, A. J.; Abbatt, J. P. D. Aqueous Photoreactions of Wood Smoke Brown Carbon. *ACS Earth Space Chem* **2020**, *4* (7), 1149–1160. https://doi.org/10.1021/acsearthspacechem.0c00117.

(7) Choudhary, V.; Roson, M. L.; Guo, X.; Gautam, T.; Gupta, T.; Zhao, R. Aqueous-Phase Photochemical Oxidation of Water-Soluble Brown Carbon Aerosols Arising from Solid Biomass Fuel Burning. *Environmental Science: Atmospheres* **2023**, *3* (5), 816–829. https://doi.org/10.1039/D2EA00151A.
